# A two-sample Mendelian randomization analysis: causal association between chemokines and pan-carcinoma

**DOI:** 10.3389/fgene.2023.1285274

**Published:** 2023-11-23

**Authors:** Kai Cui, Na Song, Yanwu Fan, Liqun Zeng, Pingyu Shi, Ziwei Wang, Wei Su, Haijun Wang

**Affiliations:** ^1^ Department of Pathology, Xinxiang Medical University, Xinxiang, China; ^2^ Department of Pathology, Xinxiang Key Laboratory of Tumor Precision Medicine, The First Affiliated Hospital of Xinxiang Medical University, Xinxiang, China

**Keywords:** causal association, pan-carcinoma, GWAS, Mendelian randomization, chemokine

## Abstract

**Objective:** According to the 2020 data from the World Health Organization (WHO), cancers stand as one of the foremost contributors to global mortality. Revealing novel cancer risk factors and protective factors is of paramount importance in the prevention of disease occurrence. Studies on the relationship between chemokines and cancer are ongoing; however, due to the coordination of multiple potential mechanisms, the specific causal association remains unclear.

**Methods:** We performed a bidirectional Mendelian randomization analysis to explore the causal association between serum chemokines and pan-carcinoma. All data is from the GWAS catalog and IEU Open GWAS database. The inverse-variance weighted (IVW) method is primarily employed for assessing the statistical significance of the findings. In addition, the significance threshold after the multiple hypothesis test (Bonferroni) was 0.0013, and the evidence of a potential association was considered if the *p*-value < 0.05, but remained greater than Bonferroni’s threshold.

**Results:** The results indicate that CCL1 (odds ratio, OR = 1.18), CCL2 (OR = 1.04), CCL8 (OR = 1.36), CCL14 (Colorectal, OR = 1.08, Small intestine, OR = 0.77, Lung, OR = 1.11), CCL15 (OR = 0.85), CCL18 (Breast, OR = 0.95, Prostate, OR = 0.96), CCL19 (Lung, OR = 0.66, Prostate, OR = 0.92), CCL20 (Lung, OR = 0.53, Thyroid, OR = 0.76), CCL21 (OR = 0.62), CCL22 (OR = 2.05), CCL23 (OR = 1.31), CCL24 (OR = 1.06), CCL27 (OR = 1.49), CCL28 (OR = 0.74), CXCL5 (OR = 0.95), CXCL9 (OR = 3.60), CXCL12 (Breast, OR = 0.87, Small intestine, OR = 0.58), CXCL13 (Breast, OR = 0.93, Lung, OR = 1.29), CXCL14 (Colon, OR = 1.40) and CXCL17 (OR = 1.07) are potential risk factors for cancers. In addition, there was a reverse causal association between CCL1 (OR = 0.94) and CCL18 (OR = 0.94) and breast cancer. Sensitivity analysis results were similar. The results of the other four MR Methods were consistent with the main results, and the leave-one-out method showed that the results were not driven by a Single nucleotide polymorphism (SNP). Moreover, there was no heterogeneity and pleiotropy in our analysis.

**Conclusion:** Based on the two-sample MR Analysis method, we found that chemokines might be upstream factors of cancer pathogenesis. These results might provide new insights into the future use of chemokines as potential targets for cancer prevention and treatment. Our results also provide important clues for tumor prevention, and changes of serum chemokine concentration may be recognized as one of the features of precancerous lesions in future clinical trials.

## Introduction

A substantial number of new cancer cases are diagnosed annually, and most of them die from the disease. A significant proportion of cancer patients, such as those with pancreatic cancer, were diagnosed at an advanced stage due to a poor prognosis, high mortality rates, and rapid disease progression (Halbrook et al., 2023). Fortunately, due to the progress and improvement of treatment methods, there has been a significant reduction in the incidence of cervical cancer among vaccinated women. Similarly, advancements in immunotherapy and targeted therapy have led to a significant reduction in mortality rates for melanoma, kidney cancer, and other types of cancer. However, the incidence of breast, uterine, and prostate cancers continues to exhibit an upward trend year after year (Siegel et al., 2023). In order to reduce the incidence of cancer, the discovery of risk factors in precancerous lesions is particularly important. So far, prospective studies have identified several factors that can interfere with cancer risk (Kliemann et al., 2023; Lagou and Karagiannis, 2023; Wang et al., 2023). For example, processed food intake and obesity can influence changes in a range of cancer risk indicators. In addition, a meta-analysis investigated the complexity of aging and cancer risk (López-Otín et al., 2023). A growing number of factors are proving to be associated with cancer risk. The discovery of risk factors may provide potential value for cancer prevention.

In recent years, more and more studies have confirmed the potential value of chemokines for cancer progression and treatment (Märkl et al., 2022; Propper and Balkwill, 2022). Chemokines are a class of cytokines that transport immune cells and are associated with lymphoid tissue (Schulz et al., 2016; Cambier et al., 2023). In cancer, however, they promoted the migration of immunosuppressive cells, such as Tregs, M2 macrophages, and so on (Moreno Ayala et al., 2023; Zhou et al., 2023). Furthermore, chemokines promoted cancer progression by mediating tumor-related pathways such as PI3K/AKT and ERK1/2 (Zhao et al., 2017). However, it should be noted that not all chemokines are implicated in tumor progression; indeed, certain chemokines exhibit anti-tumor effects (Korbecki et al., 2020). Some studies had found that high-expression chemokines are more sensitive to cancer immunotherapy (Limagne et al., 2022). Non-small cell lung cancers with high CXCL10 expression had a better response when treated with immune checkpoint suppression. In addition, the chemokine CXCL10 recruited CD4^+^ and CD8^+^ T cells to the tumor via CCR6^+^ type 3 innate lymphoid cells (Bruchard et al., 2022). Surprisingly, CXCL10 also promoted tumor cell migration in mouse models (Hirth et al., 2020), and CXCL10 secreted by mesenchymal stem cells promoted tumor growth (Timaner et al., 2018). In addition, the relationship between other chemokines and tumors is particularly complex. Curiously, if there is a causal association between chemokines and cancers. Although several meta-analyses had been conducted to explore causal associations between chemokines and cancer (Cho and Kim, 2013; Liu et al., 2018), there had not been a systematic comprehensive study.

The above studies are fuzzy about the association between chemokines and cancers, which may be influenced by environmental and other factors. Therefore, it is necessary to conduct a Mendelian randomization (MR) study between chemokines and tumors. MR uses genetic variation as instrumental variables (IVs) to measure potential causal associations between exposures and outcomes (Cheng et al., 2022). Single nucleotide polymorphisms (SNPs) were obtained from genome-wide association studies. The advantage of MR is to establish a causal association between exposures and outcomes from a genetic perspective, excluding other external environmental and confounding factors (Timaner et al., 2018). So, the association between chemokines and the risk of 14 types of malignancies were evaluated using two-sample MR Analysis in our study.

## Materials and methods

### Study design

**FIGURE 1:**
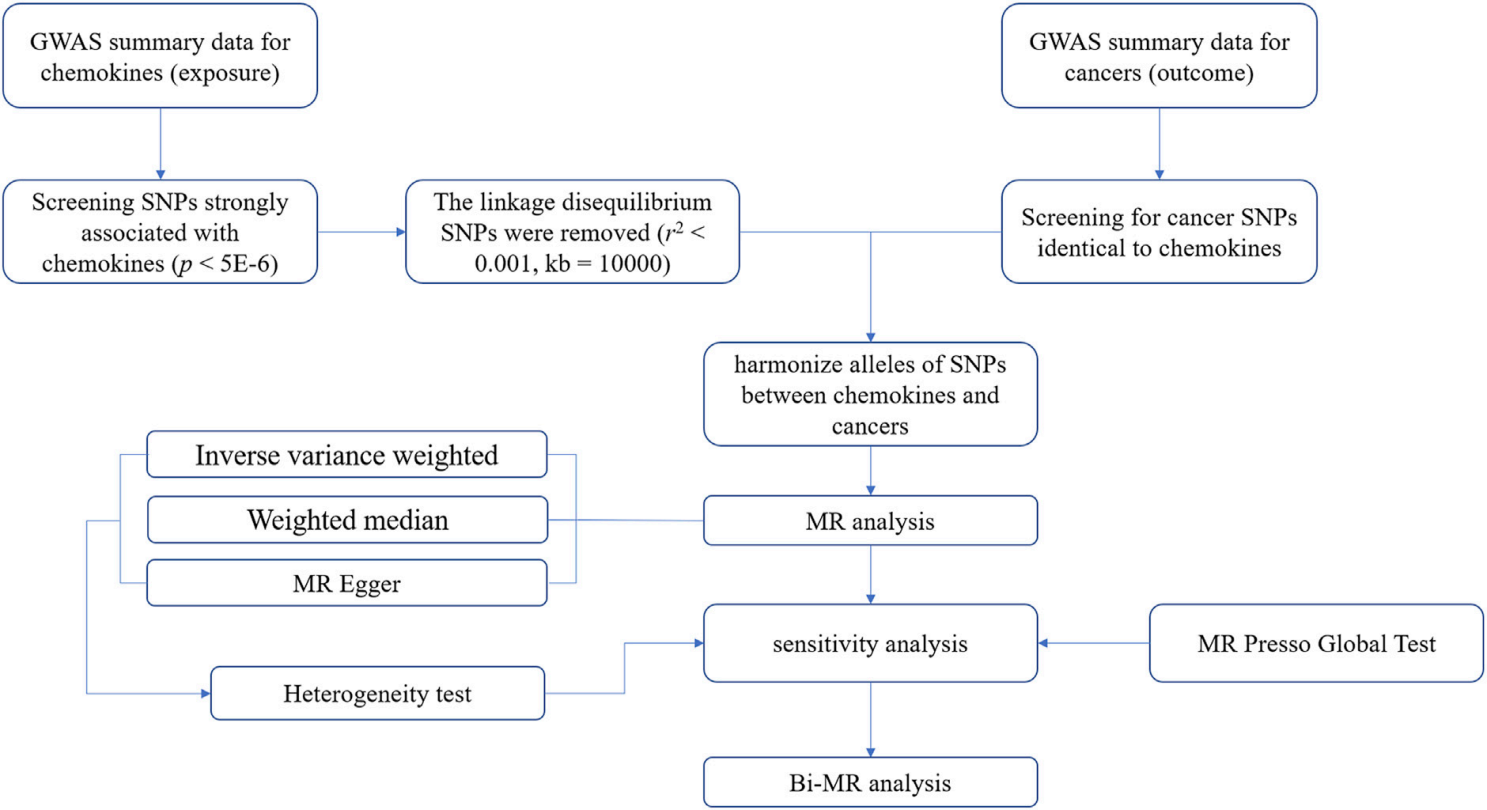
A flowchart for analyzing causal associations between chemokines and tumors based on Mendelian randomization (MR).

We conducted a two-sample MR Analysis between cancers and chemokines using publicly available online data. Including GWAS Catalog (https://www.ebi.ac.uk/gwas/) and IEU OpenGWAS (https://gwas.mrcieu.ac.uk/) (Buniello et al., 2019). These databases have received ethical approval and informed consent, so no additional instructions are required. Three preconditions must be met when performing MR analysis (Guyatt et al., 2023). First, the association hypothesis: IVs must be strongly associated with chemokines, and F-value is considered as measure indicator of association. Second, the independence hypothesis: IVs and confounding factors were independent of each other. In short, chemokines IVs were not associated with other factors that had a causal association with the tumors. Third, the exclusivity hypothesis: IVs influence tumors only through chemokines (Figure 1).

### Exposure and outcome data

Chemokines data came from a study on serum proteins in the GWAS catalog. To explore associations between genetic variants and serum proteins, Gudjonsson et al. (2022) conducted a GWAS study involving 5,368 European individuals. We downloaded 38 serum chemokine protein-associated SNPs from the study as exposure factors.

The outcome factors were 14 cancers, including breast cancer, lung cancer, gastrointestinal cancer and some other site-specific tumors. IVs for Breast Cancer were derived from the Breast Cancer Association Consortium [BCAC (Oncoarray, *N* = 106,776) (iCOGS, *N* = 89,677), FinnGen database (*N* = 123,579) (Michailidou et al., 2017; Kurki et al., 2023). IVs for prostate cancer were obtained from Schumacher’s GWAS data (*N* = 140,254) (Schumacher et al., 2018). Malignant neoplasm of ovary (*N* = 123,579) and all other tumor IVs (*N* = 218,792) were derived from the FinnGen database. All sources of tumor GWAS information are provided in the Supplementary Table S1.

### Instrumental variable selection

First, the selection of instrumental variables cannot violate the first hypothesis of Mendelian randomization, so we used the threshold of *p* < 5E-8 to screen the IVs strongly related to serum chemokines. However, some chemokines did not have SNPs with this threshold, and then the threshold of significance was eased to *p* < 5E-6 (Luo et al., 2022; Yu et al., 2023). And the SNPs with F values less than 10 were excluded (Luo et al., 2021). SNPs with F statistic >10 are considered to be strongly associated with exposure. Secondly, there may be linkage disequilibrium (LD) between SNPs. The LD phenomenon implies non-random transmission of different alleles to offspring, and it is crucial to maintain LD between various SNPs prior to conducting MR analysis (Yarmolinsky et al., 2023). To eliminate LD, the TwoSample MR package was employed in this study with specific parameters set as *r* ^2^ = 0.001 and kb = 10,000. The variable *r* ^2^ represents the association of LD between SNPs, while kb represents the region range of LD between SNPs. Third, information about the SNPs in the outcome was matched according to the SNPs screened during exposure. In this process, in the absence of SNP information, substitute proxy SNPs are not utilized. Finally, SNPs with palindromic structure were removed.

### MR analysis

To determine the causal association between serum chemokines and cancers, a two-sample MR Analysis was performed. A total of three common MR Analysis methods have been used, including inverse-variance weighted (IVW) (Huang et al., 2022), MR-Egger regression (Wu et al., 2020), weighted median (Li et al., 2022), weighted mode and simple mode methods are supplemented. According to the survey, the IVW test exhibits superior advantages compared to additional methods (Lin et al., 2021). And it has been used as the primary MR Analysis method in most studies (Yang M. et al., 2023; Yang Y. et al., 2023; Ding et al., 2023; Li et al., 2023). Similarly, IVW was used as the main test method in our study, while other methods were used as references. In addition, the MR-Egger regression test and MR-Presso were used to verify the existence of horizontal pleiotropy, and *p*-value < 0.05 is considered to be horizontal pleiotropy. To ensure the validity of our findings, we conducted leave-one-out sensitivity analysis to ascertain whether a single SNP is responsible for driving the results. Based on the causal relationship between 38 chemokines and cancer, the more conservative Bonferroni method was used to correct for significance results. Before correction, *p* < 0.05 was a significant result, and after correction, *p* < 0.0013 was a significant result. Results with *p* < 0.05 but higher than 0.0013 were considered for potential causal associations (Sedgwick, 2014; Larsson et al., 2017). All statistical tests were performed in two-sample MR and MR-PRESSO packages. Moreover, Heterogeneity test results were significant (*p* < 0.05), which was considered to be heterogeneity among IVs.

To investigate the bidirectional causal relationship between cancer and chemokines, we performed a bi-directional Mendelian randomization (bi-MR) analysis. Cancers were used as exposure variable and chemokines as outcome variable.

## Result

### SNP data

First, significant SNPs were screened by *p*-value. Some chemokines did not detect SNPs with *p*-values less than 5E-8, in addition, CCL24 had less than 3 SNPs below this threshold. So, a significance threshold of 5E-6 was set. After significance screening (*p* < 5E-6) and LD filtering (*r* ^2^ = 0.001, kb = 10,000), a total of 828 SNPs of serum chemokine proteins were obtained. F values of 828 SNPs were calculated, and the values were > 10, suggesting that there was no weak instrument bias. Information on all SNPs with a threshold of 5E-6 was shown in Supplementary Table S2 (including F values). IVW test was used as the main MR Analysis method for all chemokines. The statistical results between 38 chemokines and pan-carcinoma were shown in Supplementary Table S3. Similar results were obtained for all sensitivity analyses. The results of heterogeneity analysis and pleiotropy analysis were shown in Supplementary Table S4.

Bi-MR Analysis was performed for all results that met the significance threshold. To ensure sufficient SNPs were available for MR Analysis, the SNPs threshold was set at 5E-8 for breast cancer (excluding finn-b-C3_BREAST) and prostate cancer, and 5E-6 for other malignancies.

In addition, for the results of significance, the *p*-values of the heterogeneity test were all > 0.05 and there was no pleiotropy, Including MR-egger and MR-Presso methods. Moreover, the leave-one-out sensitivity analysis did not find that causality was determined by a single SNP (Supplementary Table S5).

### Breast cancer

**FIGURE 2:**
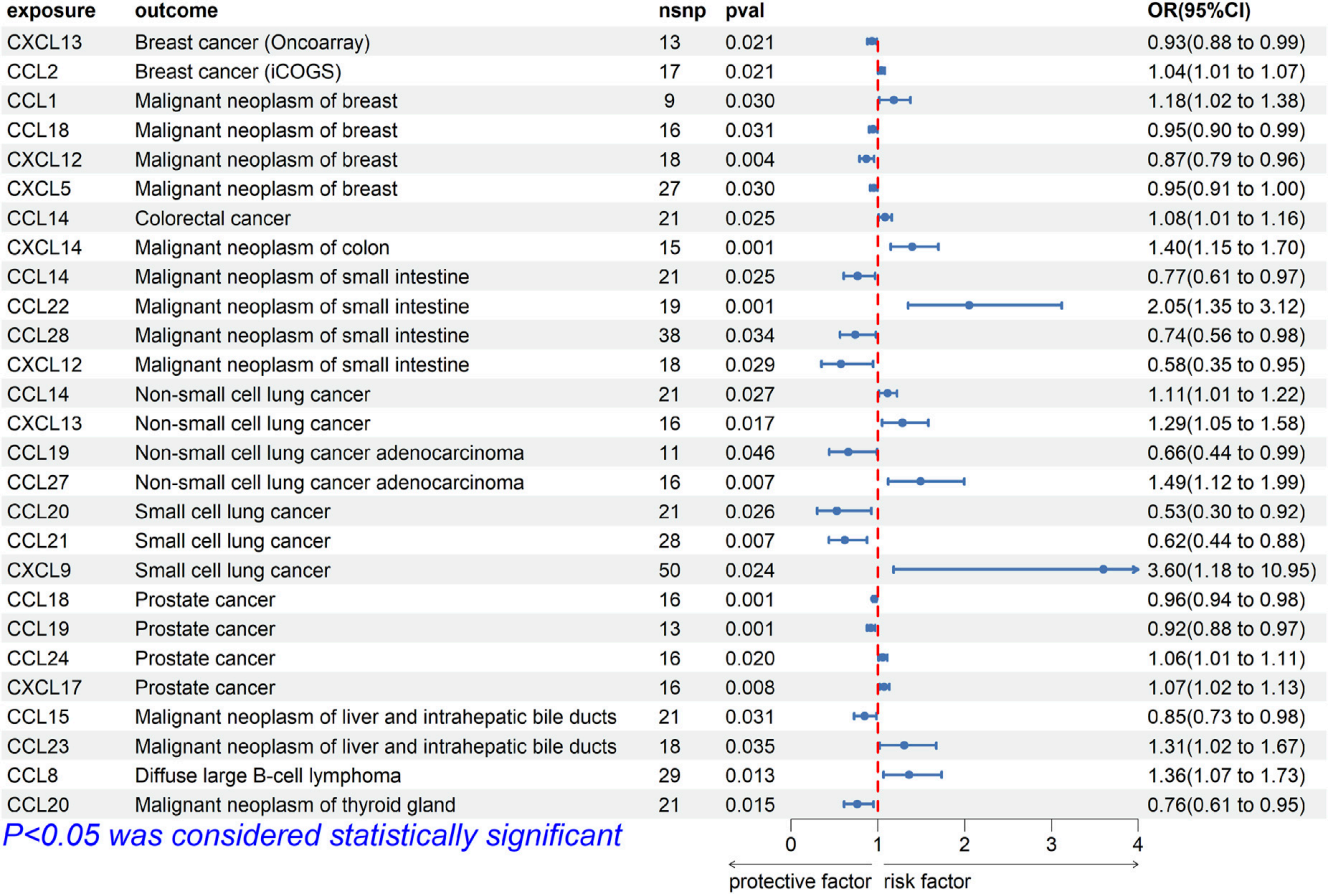
Forest plot for the causal association of chemokines on the risk of tumors derived from IVW. The OR value > 0 is considered a risk factor for tumor. The OR value < 0 is considered a protective factor for tumor. OR, odds ratio; CI, confidence interval.

For breast cancer, we investigated the causal association between chemokines and the disease using three breast cancer GWAS datasets. The results of MR analysis showed significant causal association between CXCL13 [OR (95%CI), 0.93 (0.88–0.99), *p* = 0.021] and breast cancer (ieu-a-1129), CCL2 [OR (95%CI), 1.04 (1.01–1.07), *p* = 0.021] and breast cancer (ieu-a-1130), and CCL1 [OR, (95%CI), 1.18 (1.02–1.38), *p* = 0.030], CCL18 [OR (95%CI), 0.95 (0.90–0.99), *p* = 0.031], CXCL5 [OR (95%CI), 0.95 (0.91–1.00), *p* = 0.030], CXCL12 [OR (95%CI), 0.87 (0.79–0.96), *p* = 0.004] and breast cancer (finn-b-C3_BREAST) (Figure 2). The results were not consistent across different GWAS data, which might be due to different IVs.

Cochrane’s Q test did not provide evidence of heterogeneity between CCL1 (*p* = 0.227), CCL2 (*p* = 0.982), CCL18 (*p* = 0.221), CXCL5 (*p* = 0.533), CXCL12 (*p* = 0.977), and CXCL13 (*p* = 0.520) and breast cancer. The intercept of MR-Egger test did not detect pleiotropy of SNPs for CCL1 (*p* = 0.779), CCL2 (*p* = 0.519), CCL18 (*p* = 0.141), CXCL5 (*p* = 0.639), CXCL12 (*p* = 0.800) and CXCL13 (*p* = 0.643). The MR-Presso test did not detect abnormal SNPs and there was no pleiotropy between SNPs (CCL1 *p* = 0.223, CCL2 *p* = 0.943, CCL18 *p* = 0.413, CXCL5 *p* = 0.497, CXCL12 *p* = 0.968, CXCL13 *p* = 0.569). These results suggest that the serum proteins CCL18, CXCL5, CXCL12, and CXCL13 are protective factors for breast cancer, while CCL1 and CCL2 are risk factors for breast cancer.

### Intestinal cancer

For intestinal cancer, we investigated the causal association between chemokines and the disease. The results of MR analysis showed that significant causal association between CCL14 [OR (95%CI), 1.083 (1.010–1.161), *p* = 0.03] and colorectal cancer (finn-b-C3_COLORECTAL), CXCL14 [OR (95%CI), 1.397 (1.150–1.698), *p* = 7.98E-04] and colon cancer (finn-b-C3_COLON), CCL22 [OR (95%CI), 2.051 (1.350–3.116), *p* = 7.58E-04], CCL28 [OR (95%CI), 0.741 (0.562–0.977), *p* = 0.03], CCL14 [OR (95%CI), 0.766 (0.607–0.967), *p* = 0.03] and CXCL12 [OR (95%CI), 0.576 (0.351–0.946), *p* = 0.03] and small intestinal malignant neoplasm (finn-b-C3_SMALL_INTESTINE) (Figure 2).

Cochrane’s Q test did not provide evidence of heterogeneity between CCL14 (*p* = 0.768, *p* = 0.808), CCL22 (*p* = 0.502), CCL28 (*p* = 0.175), CXCL12 (*p* = 0.530) and CXCL14 (*p* = 0.534) and intestinal cancer. The intercept of MR-Egger test did not detect pleiotropy of SNPs for CCL14 (*p* = 0.713, *p* = 0.399), CCL22 (*p* = 0.220), CCL28 (*p* = 0.278), CXCL12 (*p* = 0.938) and CXCL14 (*p* = 0.867). The MR-Presso test did not detect abnormal SNPs and there was no pleiotropy between SNPs (CCL14 *p* = 0.858, *p* = 0.817, CCL22 *p* = 0.577, CCL28 *p* = 0.182, CXCL12 *p* = 0.549, CXCL14 *p* = 0.547). These results suggest that the serum proteins CCL14 is a protective factor for colorectal cancer, CXCL14 is a risk factor for colon cancer, CCL14, CCL28 and CXCL12 are protective factors for malignant neoplasm of small intestine, while CCL22 is a risk factor for malignant neoplasm of small intestine.

### Lung cancer

For lung cancer, we investigated the causal association between chemokines and the disease. The results of MR analysis showed that significant causal association between CCL14 [OR (95%CI), 1.111 (1.018–1.220), *p* = 0.03] and CXCL13 [OR (95%CI), 1.286 (1.047–1.579), *p* = 0.02] and non-small cell lung cancer (finn-b-C3_LUNG_NONSMALL), CCL27 [OR (95%CI), 1.493 (1.118–1.994), *p* = 0.007] and CCL19 [OR (95%CI), 0.660 (0.438–0.993), *p* = 0.046] and adenocarcinoma (finn-b-C3_NSCLC_ADENO), CXCL9 [OR (95%CI), 3.597 (1.182–10.953), *p* = 0.02], CCL20 [OR (95%CI), 0.527 (0.300–0.925), *p* = 0.03], and CCL21 [OR (95%CI), 0.619 (0.438–0.877), *p* = 0.01] and small cell lung cancer (finn-b-C3_SCLC) (Figure 2).

Cochrane’s Q test did not provide evidence of heterogeneity between CCL14 (*p* = 0.916), CCL19 (*p* = 0.446), CCL20 (*p* = 0.130), CCL21 (*p* = 0.878), CCL27 (*p* = 0.762), CXCL9 (*p* = 0.340) and CXCL13 (*p* = 0.770) and lung cancer. The intercept of MR-Egger test did not detect pleiotropy of SNPs for CCL14 (*p* = 0.247), CCL19 (*p* = 0.696), CCL20 (*p* = 0.732), CCL21 (*p* = 0.373), CCL27 (*p* = 0.963), CXCL9 (*p* = 0.455) and CXCL13 (*p* = 0.686). The MR-Presso test did not detect abnormal SNPs and there was no pleiotropy between SNPs (CCL14 *p* = 0.923, CCL19 *p* = 0.481, CCL20 *p* = 0.124, CCL21 *p* = 0.898, CCL27 *p* = 0.764, CXCL9 *p* = 0.239, CXCL13 *p* = 784). These results suggest that the serum proteins CCL14, CCL27, and CXCL13 are risk factors for non-small cell lung cancer, while CCL19 is a protective factor for non-small cell lung cancer, CXCL9 is a risk factor for small cell lung cancer, while CCL20 and CCL21 are protective factors for small cell lung cancer.

### Other cancer

For prostate cancer, we investigated the causal association between chemokines and the disease. The results of MR analysis showed that significant causal association between CCL18 [OR (95%CI), 0.961 (0.939–0.984), *p* = 1.13E-03], CCL19 [OR (95%CI), 0.920 (0.875–0.968), *p* = 1.20E-03], CCL24 [OR (95%CI), 1.058 (1.009–1.109), *p* = 0.02] and CXCL17 [OR (95%CI), 1.074 (1.020–1.132), *p* = 7.83E-03] and prostate cancer (ebi-a-GCST006085) (Figure 2).

Cochrane’s Q test did not provide evidence of heterogeneity between CCL18 (*p* = 0.767), CCL19 (*p* = 0.093), CCL24 (*p* = 0.935), CXCL17 (*p* = 0.210) and prostate cancer. The intercept of MR-Egger test did not detect pleiotropy of SNPs for CCL18 (*p* = 0.429), CCL19 (*p* = 0.790), CCL24 (*p* = 0.886) and CXCL17 (*p* = 0.886). The MR-Presso test did not detect abnormal SNPs and there was no pleiotropy between SNPs (CCL18 *p* = 657, CCL19 *p* = 0.090, CCL24 *p* = 0.937, CXCL17 *p* = 0.228). These results suggest that the serum proteins CCL24, CXCL17 are risk factors for prostate cancer, while CCL18 and CCL19 are protective factors for prostate cancer.

For liver cancer, we investigated the causal association between chemokines and the disease. The results of MR analysis showed that significant causal association between CCL15 [OR (95%CI), 0.848 (0.730–0.985), *p* = 0.03] and CCL23 [OR (95%CI), 1.306 (1.020–1.673), *p* = 0.03] and malignant neoplasm of liver (finn-b-C3_LIVER_INTRAHEPATIC_BILE_DUCTS) (Figure 2).

Cochrane’s Q test did not provide evidence of heterogeneity between CCL15 (*p* = 0.698) and CCL23 (*p* = 0.978) and liver cancer. The intercept of MR-Egger test did not detect pleiotropy of SNPs for CCL15 (*p* = 0.440) and CCL23 (*p* = 0.672). The MR-Presso test did not detect abnormal SNPs and there was no pleiotropy between SNPs (CCL15 *p* = 0.784, CCL23 *p* = 0.994). These results suggest that the serum proteins CCL23 is a risk factor for malignant neoplasm of liver, while CCL15 is a protective factor for malignant neoplasm of liver.

For Diffuse large B-cell lymphoma (DLBL), we investigated the causal association between chemokines and the disease. The results of MR analysis showed that significant causal association between CCL8 [OR (95%CI), 1.360 (1.065–1.734), *p* = 0.01] and Diffuse large B-cell lymphoma (finn-b-C3_DLBCL) (Figure 2). Cochrane’s Q test did not provide evidence of heterogeneity between CCL8 (*p* = 0.293) and Diffuse large B-cell lymphoma. The intercept of MR-Egger test did not detect pleiotropy of SNPs for CCL8 (*p* = 0.099). The MR-Presso test did not detect abnormal SNPs and there was no pleiotropy between SNPs (*p* = 0.286). These results suggest that the serum proteins CCL8 is a risk factor for DLBL, For thyroid cancer, we investigated the causal association between chemokines and the disease. The results of MR analysis showed that significant causal association between CCL20 [OR (95%CI), 0.763 (0.614–0.949), *p* = 0.02] and Malignant neoplasm of thyroid gland (finn-b-C3_THYROID_GLAND) (Figure 2). Cochrane’s Q test did not provide evidence of heterogeneity between CCL20 (*p* = 0.357) and thyroid cancer. The intercept of MR-Egger test did not detect pleiotropy of SNPs for CCL20 (*p* = 0.887). The MR-Presso test did not detect abnormal SNPs and there was no pleiotropy between SNPs (*p* = 0.442). These results suggest that the serum proteins CCL20 is a protective factor for malignant neoplasm of thyroid gland.

In addition, the causal association between chemokines and other tumors had also been analyzed, such as malignant tumors of the brain, stomach, pancreas, kidney, ovary, skin, and acute lymphoblastic leukemia. However, there was no causal association between them.

### Bi-causal effects between chemokines and tumor risk

**FIGURE 3:**
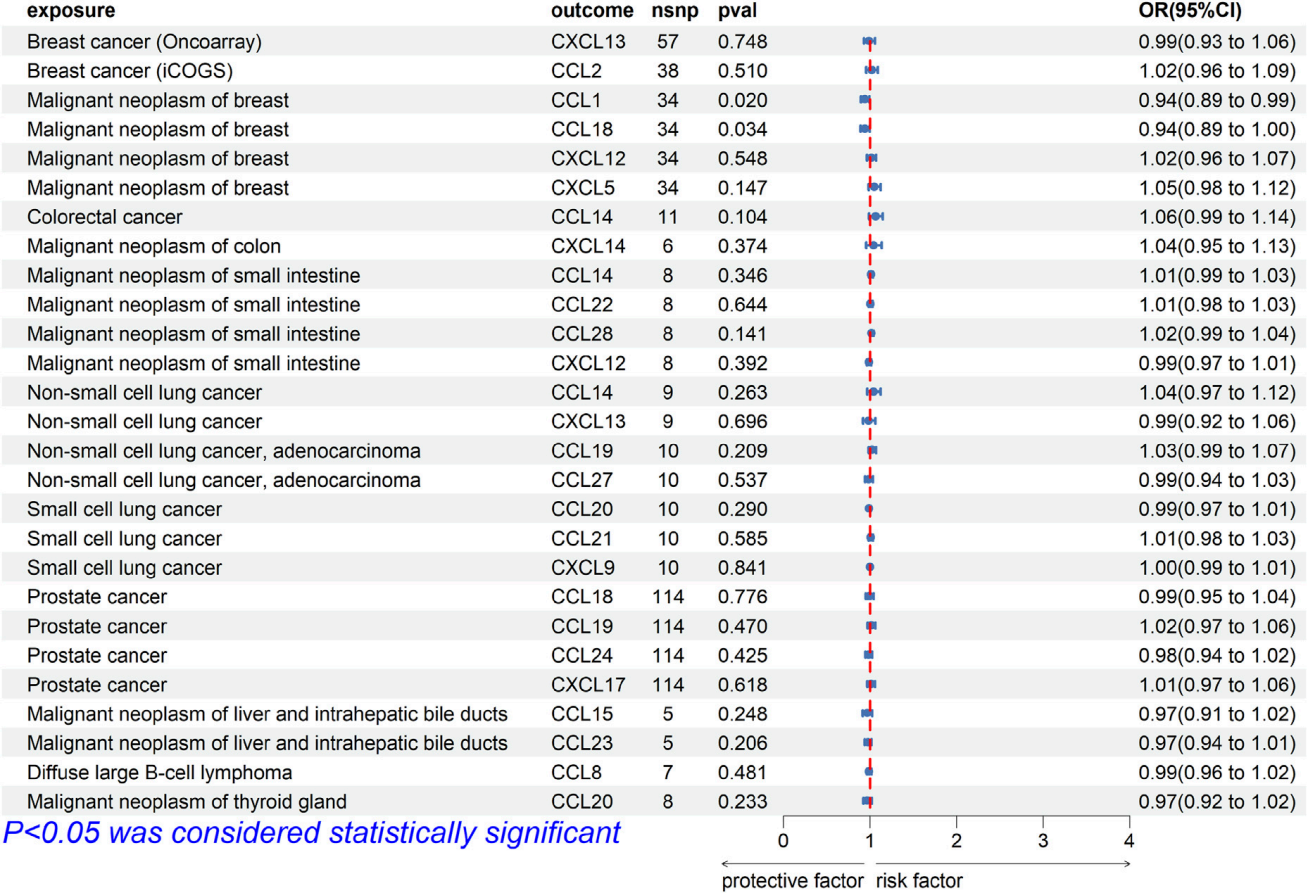
Forest plot for the reverse causal association of chemokines on the risk of tumors derived from IVW. The OR value > 0 is considered a risk factor for tumor. The OR value < 0 is considered a protective factor for tumor. OR, odds ratio; CI, confidence interval.

To explore whether there was reverse causality in the significant results obtained, we regarded cancer as the exposure factor, chemokines as the outcome, and cancer-related SNPs (*p* < 5E-8 or *p* < 5E-6) as the IVs. In bi-MR, the causal association between CCL1 [OR (95%CI), 0.94 (0.89–0.99), *p* = 0.020] and CCL18 [OR (95%CI), 0.94 (0.89–1.00), *p* = 0.034] and breast cancer (finn-b-C3_BREAST) was found (Figure 3). Cochrane’s Q test did not provide evidence of heterogeneity between CCL1 (*p* = 0.675) and CCL18 (*p* = 0.336) and breast cancer. The intercept of MR-Egger test did not detect pleiotropy of SNPs for CCL1 (*p* = 0.382) and CCL18 (*p* = 0.964). The MR-Presso test did not detect abnormal SNPs and there was no pleiotropy between SNPs (CCL1 *p* = 0.595, CCL18 *p* = 0.297). In addition, MR Analysis showed no causal association between other significance result (*p* > 0.05).

## Discussion

This was the first comprehensive MR analysis to investigate the causal association between chemokines and pan-carcinoma. In two-sample MR Analysis, we initially investigated the causal association between CCL and CXC chemokines and breast, intestinal, lung, and other cancers. Based on the genetic variation of serum protein chemokines and cancers in the publicly available database, it was found that causal association between chemokines and cancer susceptibility. Interestingly, there were also causal association between cancers and partial chemokines. These clues suggest that some chemokines are upstream to drive or hinder the development of cancers.

In our results, there was causal association between 20 chemokines and cancers, as shown in Figure 2. Some studies on chemokines were consistent with our findings. For CCL chemokines, previous studies had shown that CCL1 mainly recruits Tregs to change the tumor microenvironment and promote the progression of breast cancer stem cells (Xu et al., 2017; Kuehnemuth et al., 2018). Surprisingly, there was causal association between CCL1 and breast cancer in bi-MR analysis. The immunosuppressive mechanism of CCL1-recruited Tregs had been widely recognized, but Tregs were also key regulators of CD8^+^ T cells initiation (Pace et al., 2012). In addition, tissue-resident memory T cells were marker of good prognosis for early triple-negative breast cancer (Byrne et al., 2020). At the same time, other studies had shown that CCL1 was also present in human memory CD8 T cells (Brinza et al., 2016). Although CCL1 promotes tumor progression through Tregs, Tregs may also trigger the accumulation of CD8^+^ T cells. It could be concluded that CCL1 was upstream of breast cancer, and that breast cancer might also act on CCL1 through negative feedback. For other chemokines, according to recent research reports, CCL2 recruits monocytes to generate vascular endothelial growth factors, thereby facilitating breast cancer cell extravasation (Qian et al., 2011). There was a potential association between CCL8 and DLBL, where CCL8 was involved in the polarization of M2 macrophages and affected patient survival (Lou et al., 2022). Both CCL22 and CCL23 were immunosuppressive chemokines derived from macrophages, which had a unique role in inhibiting anti-tumor immunity (Kamat et al., 2022; Lecoq et al., 2022). In addition, CCL24 was involved in the biological process of cancer through various functions such as angiogenesis and M2 macrophage polarization (Lim, 2021). Moreover, there were some evidences that CCL27 was associated with development of tumors (Martínez-Rodríguez and Monteagudo, 2021). As shown in MR Results, CCL1 and CCL2 were risk factors for breast cancer. CCL8 was a risk factor for DLBL. CCL22 was a risk factor for small intestine malignancy. CCL23 was a risk factor for liver and bile duct malignancy. CCL24 was a risk factor for prostate cancer, and CCL27 was a risk factor for non-small cell lung cancer. Interestingly, CCL14 was a risk factor in lung cancer and colorectal cancer and a protective factor in small intestine tumors. As a chemokine that activates immune cells, studies had found that CCL14 was strongly correlated with a variety of anti-tumor immune cells, including CD8^+^ T cells, in cancers (Gu et al., 2020). However, other studies had shown that the CCL14 chemokine signaling pathway promotes cancer progression, and inhibiting the expression of CCL14 could reduce the ability of breast cancer to metastasize (Li et al., 2011). Therefore, CCL14 might have different causal associations between different cancers.

Besides, six factors had inverse causal associations with cancer, including CCL15, CCL18, CCL19, CCL20, CCL21, and CCL28. The study found that the chemokine CCL15 recruits CCR^+^ CD14^+^ monocytes in hepatocellular carcinoma, driving multiple tumor-promoting factors (Liu et al., 2019). In addition, CCL18 had been reported as a cancer risk factor in both breast and prostate cancer (Chen et al., 2011; Xu et al., 2014). However, there was an inverse association between CCL15 and CCL18 and cancers in our analysis. For other chemokines, the study had demonstrated that CCL19 exerts a potential stimulatory effect on the response of CD8^+^ T cells (Yan et al., 2021). In non-small cell lung cancer, CCL19 and CXCL11 reduced the receptor activator of nuclear factor-κB ligand/osteoprotegerin ratio, an indicator of osteoclast stimulation (Kim et al., 2015). In addition, CCL19 and CCL21 migrated dendritic cells in prostate cancer to inhibit cancer progression (Youlin et al., 2018). The same trend was found in our analysis. CCL19 was a protective factor for prostate and lung cancer. The roles of CCL20 and CCL28 in small intestinal and thyroid cancer remain insufficiently investigated, while our findings demonstrate their potential as protective factors, which might provide valuable insights for future research endeavors.

Among CXC chemokines, the causal association with cancer had three positive factors and two negative factors. CXCL9 derived Th1 responses and limited Th2 infiltration, and it was associated with favorable prognosis in small cell lung cancer (Yang L. et al., 2023), however, other studies have reported that CXCL9 binds to CXCR3 in tumors to promote EMT and cancer cell migration (Neo and Lundqvist, 2020). In addition, multiple meta-analyses showed that CXCL12 expression improved the prognosis of breast cancer patients, which was consistent with our results that CXCL12 had a reverse causal association with breast cancer (Samarendra et al., 2017; Liu et al., 2018). Moreover, the studies had shown that CXCL13 drives an anti-tumor immune response to limit tumor progression in mouse breast cancer cells (Ma et al., 2021). And TFH cells that produce CXCL13 played a key role in reversing the immunosuppressive environment induced by Tregs (Gu-Trantien et al., 2017). However, another study suggested that CXCL13 may inhibit tumor growth in breast cancer through CXCR5/ERK signaling (Xu et al., 2018). Simultaneously, in the context of lung cancer, CXCL13 was considered to be a carcinogenic cytokine with significantly enhanced expression levels and facilitating cancer cell invasion through the epithelial-mesenchymal transition (EMT) process (Kazanietz et al., 2019). In our results, CXCL13 was a protective factor in breast cancer and a risk factor in lung cancer. The expression of CXCL14 in colorectal cancer tissues was correlated with TNM stage and poor prognosis. In addition, the invasion ability of cancer cells was also regulated by CXCL14 expression (Zeng et al., 2013), which suggests the pathogenicity of CXCL14 in colorectal cancer. One study reported ventral prostate hyperplasia in estrogen receptor β−/− mice with a possible increased incidence of prostate cancer, while genetic analysis found a significant increase in CXCL17 (Wu et al., 2017). Therefore, CXCL17 might also be a potential carcinogen of prostate cancer.

Based on previous studies, some of our results were supported, but partial studies were not consistent with our results, for example, the causal association of CCL15 and CCL18 with tumors incidence. In cancer, the cancer-promoting mechanism of CCL15 was mainly dependent on the monocytes it recruits, and MR Analysis was to analyze the causal association between CCL15 and cancer alone, without involving other factors. The cancer-promoting effect of monocytes recruited by CCL15 might mask the causal association between CCL15 and cancer. In addition, CCL18 mainly recruits Tregs, Th2 and immunosuppressive cells. The effect of immunosuppressive effects on tumors may be larger than the causal association between CCL18 and cancer. In our study, the chemokine concentrations we used were located in the serum, and different locations of cytokine proteins might also cause different causal associations.

In previous analyses, there had not been a comprehensive study to analyze the causal association between chemokines and cancer. One of our strengths is to extract the genetic variation of CCL and CXC chemokines and cancers from a public database for MR Analysis. Based on our analysis, a variety of chemokines were risk factors and protective factors for cancers, and there was no heterogeneity and pleiotropy. Sensitivity analysis also obtained similar results, indicating that our results are credible and accurate. Despite the inherent advantage in MR analysis, it was important to acknowledge its limitations as well. First, we only analyzed the GWAS data of chemokines in serum, and did not analyze the chemokine concentrations in other liquid/tissue samples, which might be biased due to different sites. Secondly, chemokine and cancer GWAS data were obtained from publicly available databases, and subgroup analyses were not possible due to the lack of detailed clinical patient information. Third, the GWAS data are from European populations, and the results may not apply to non-European populations. Finally, the results of this study should still be treated with caution, and more investigations and studies should be conducted to verify the results and consider their application to clinical trial diagnosis.

## Conclusion

In summary, since the causal association between chemokines and cancer remains uncertain, and there had not been a comprehensive study to analyze the causal association between chemokines and cancer in previous studies, hereon, we performed a comprehensive two-sample MR Analysis.

As mentioned above, our results showed that causal associations of some chemokines were consistent with previous studies, including CCL2, CCL14, CCL27, CCL19, CCL21, CXCL13, CXCL14 and CXCL17. These chemokines possess the potential to serve as serum diagnostic markers. However, a large number of clinical trials are needed to verify them. In addition, some results were interesting. CCL23 was a risk factor in liver cancer and a protective factor in biliary tract cancer. In colorectal cancer, CCL14 was a risk factor, while in small intestine tumors it was a protective factor. In addition, as widely cognitive tumor-suppressor factor, CXCL9 in small cell lung cancer might be a risk factor. Further study of the underlying mechanisms of these chemokines may provide new insights into targeted therapies for tumors. Moreover, our results also provided new potential targets for tumors, including CCL8, CCL20, CCL28 and CXCL12.

Chemokines in MR results might contribute to tumor prevention and targeted therapy. At present, the detection of serum and plasma markers is crucial for cancer prevention and diagnosis. Based on our results, the serum chemokine concentrations may become new serum markers and parts of chemokines may become the potential therapy targets. Therefore, our results might provide new insights into the future use of chemokines as potential targets for cancer prevention and treatment.

## Data Availability

The original contributions presented in the study are included in the article/Supplementary Material, further inquiries can be directed to the corresponding authors.

## Supplementary material

Supplementary Table S1 Overview of the source of cancer data.

Supplementary Table S2 Overview of chemokine SNP data.

Supplementary Table S3 All results of Mendelian randomization analysis between chemokines and tumors.

Supplementary Table S4 Heterogeneity analysis and pleiotropy analysis.

Supplementary Table S5 Mendelian randomization leave-one-out sensitivity analysis.
