# Supplementary figures and images for: A two-sample Mendelian randomization analysis: causal association between chemokines and pan-carcinoma
Source: Front Genet. 2023 Nov 23;14:1285274. doi: 10.3389/fgene.2023.1285274 (PMC10702354; doi:10.3389/fgene.2023.1285274)

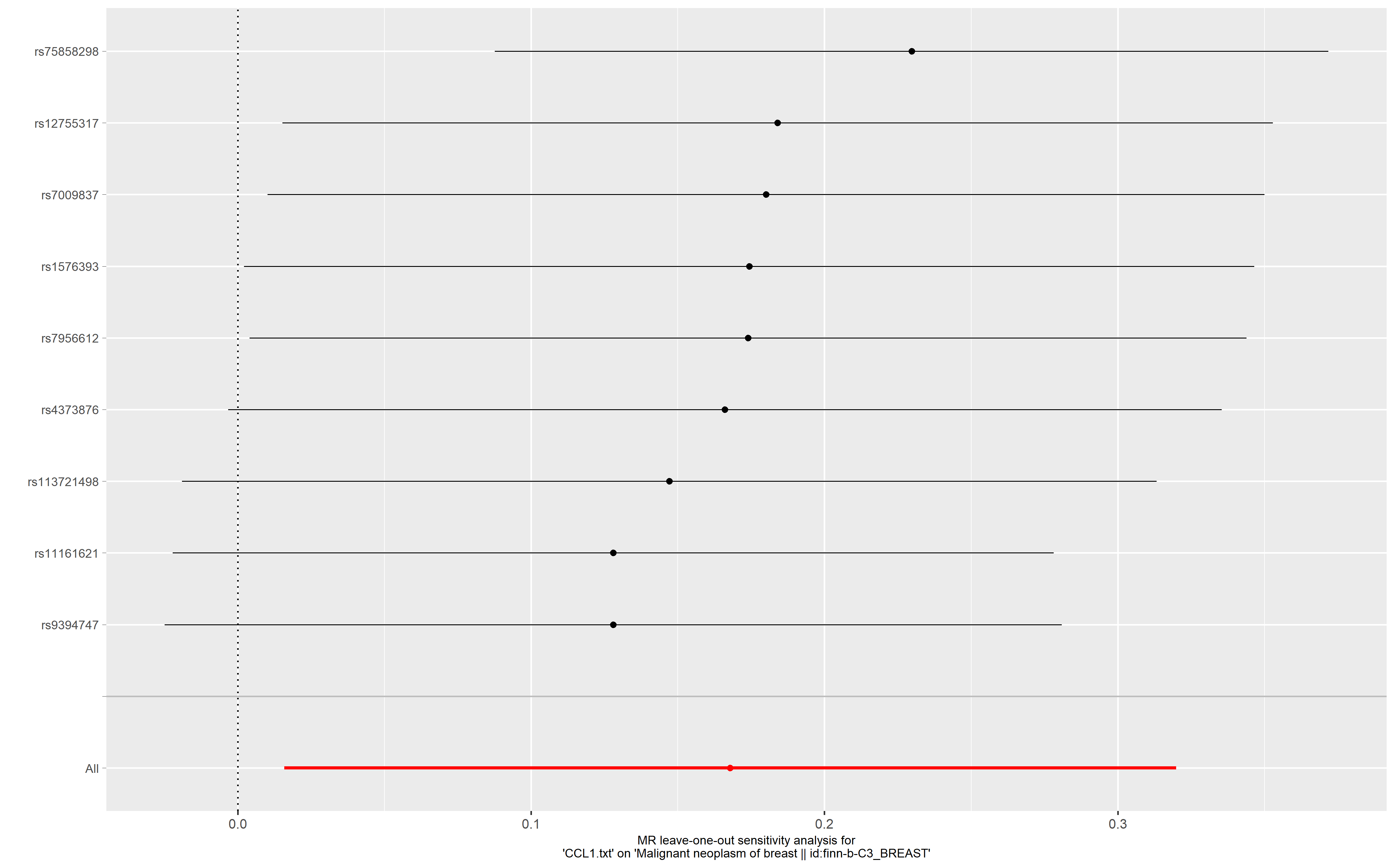


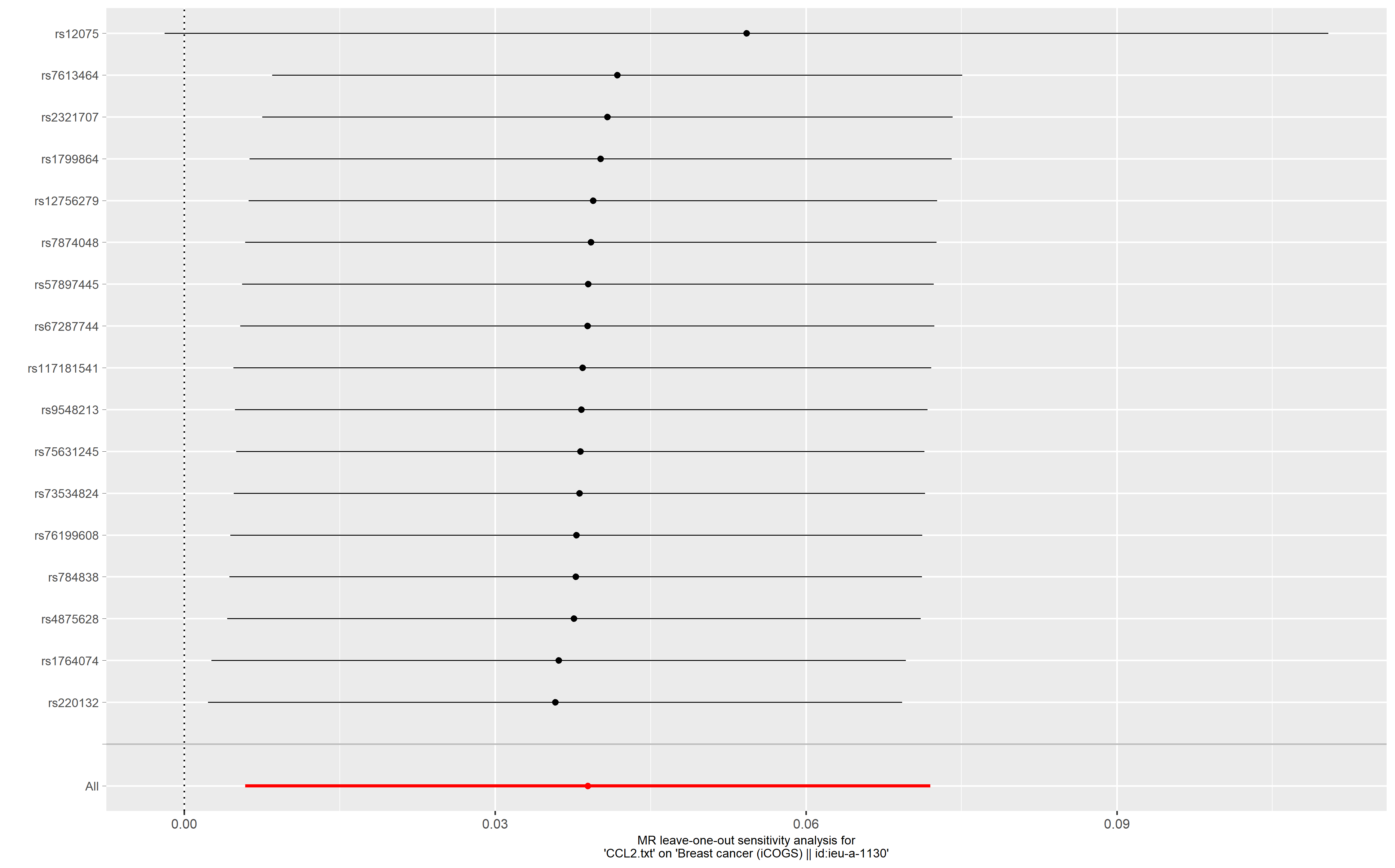


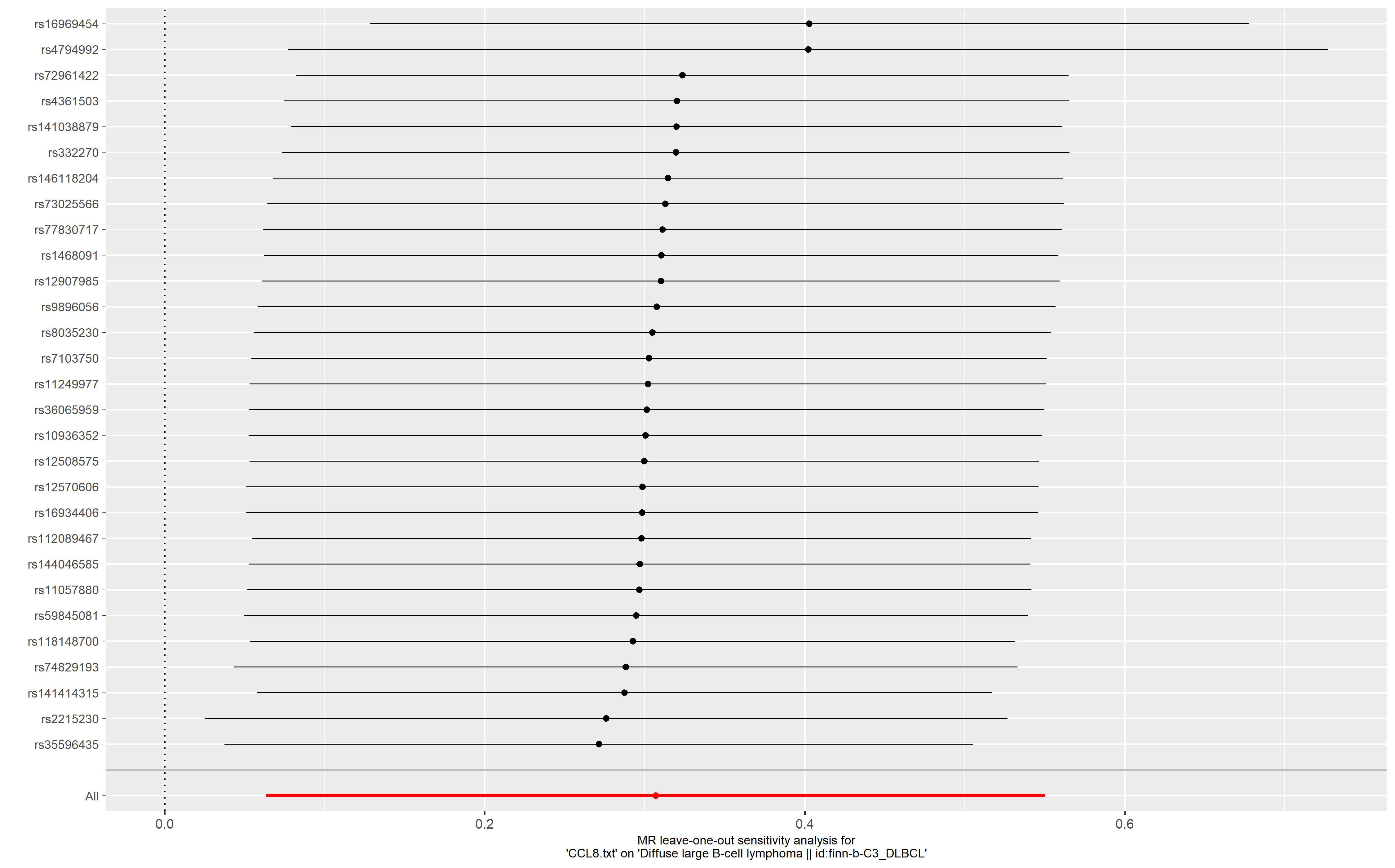


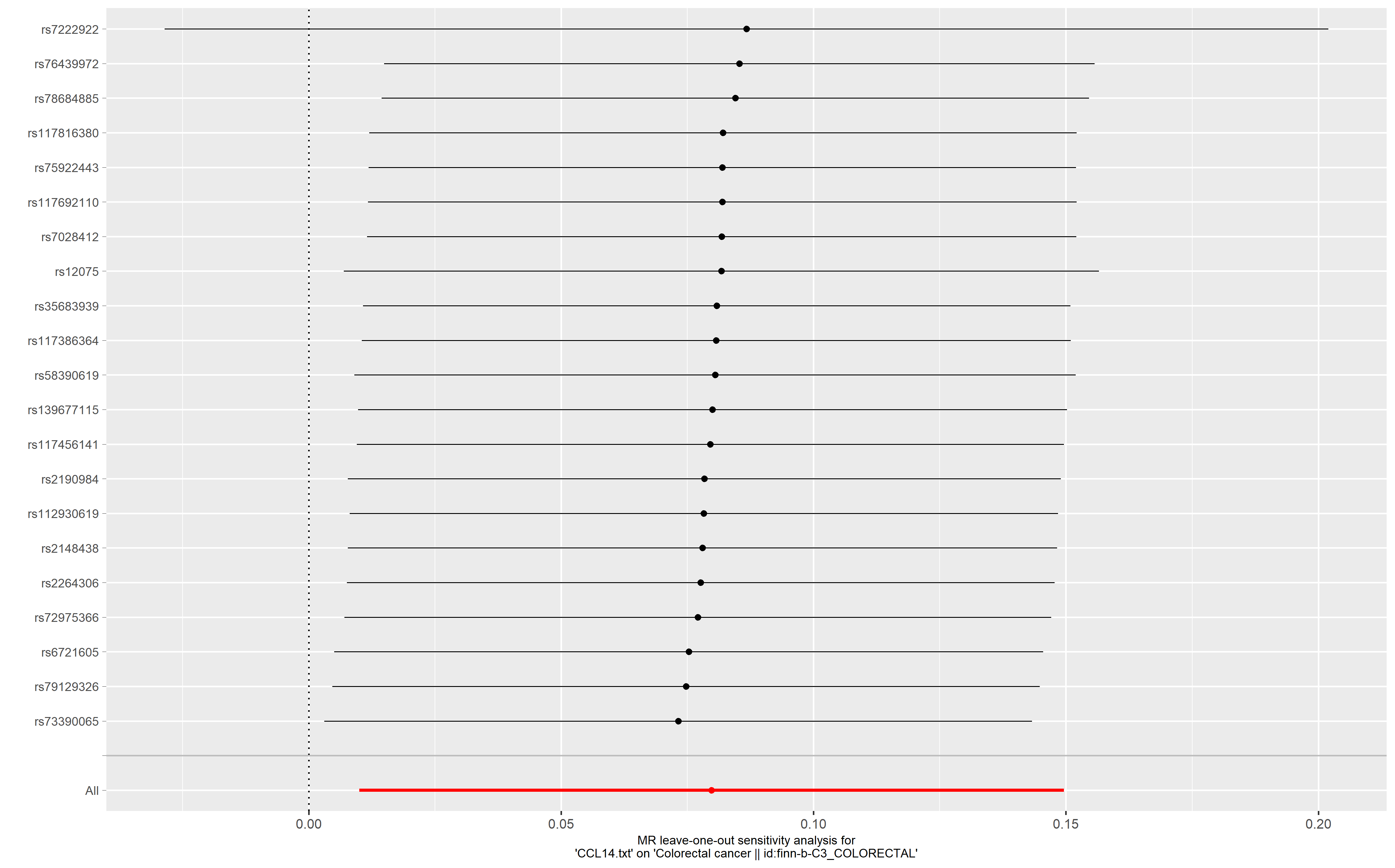


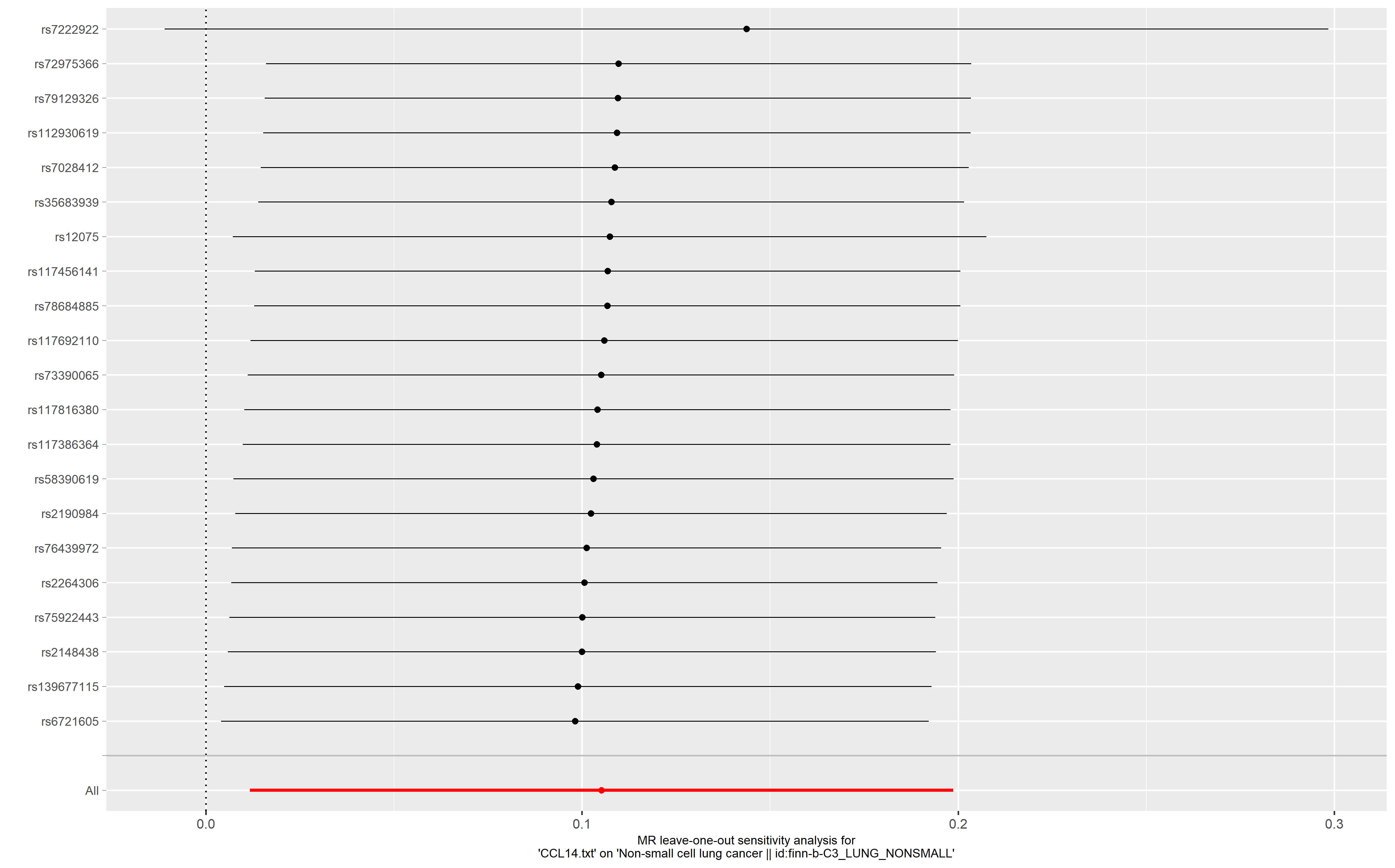


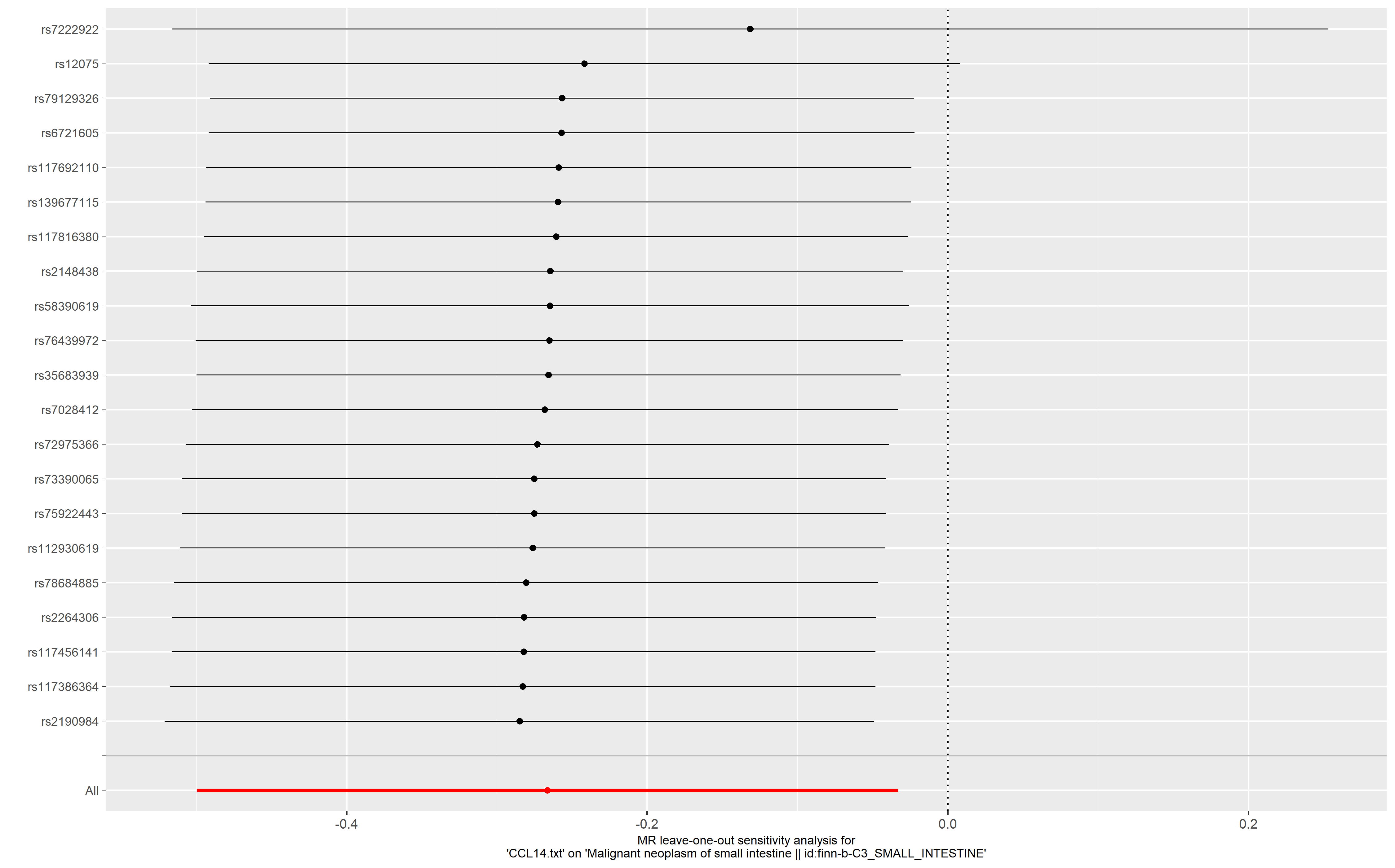


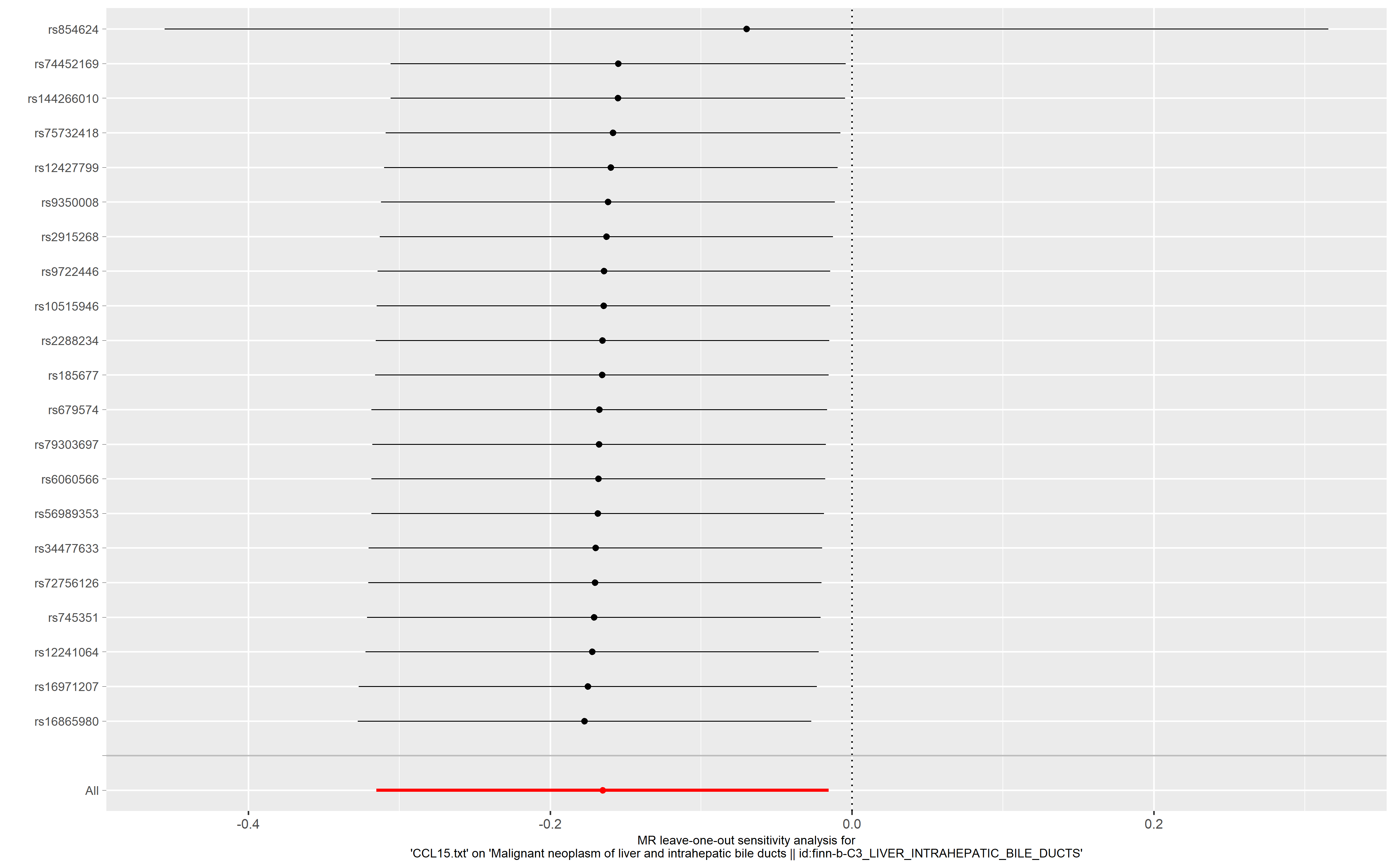


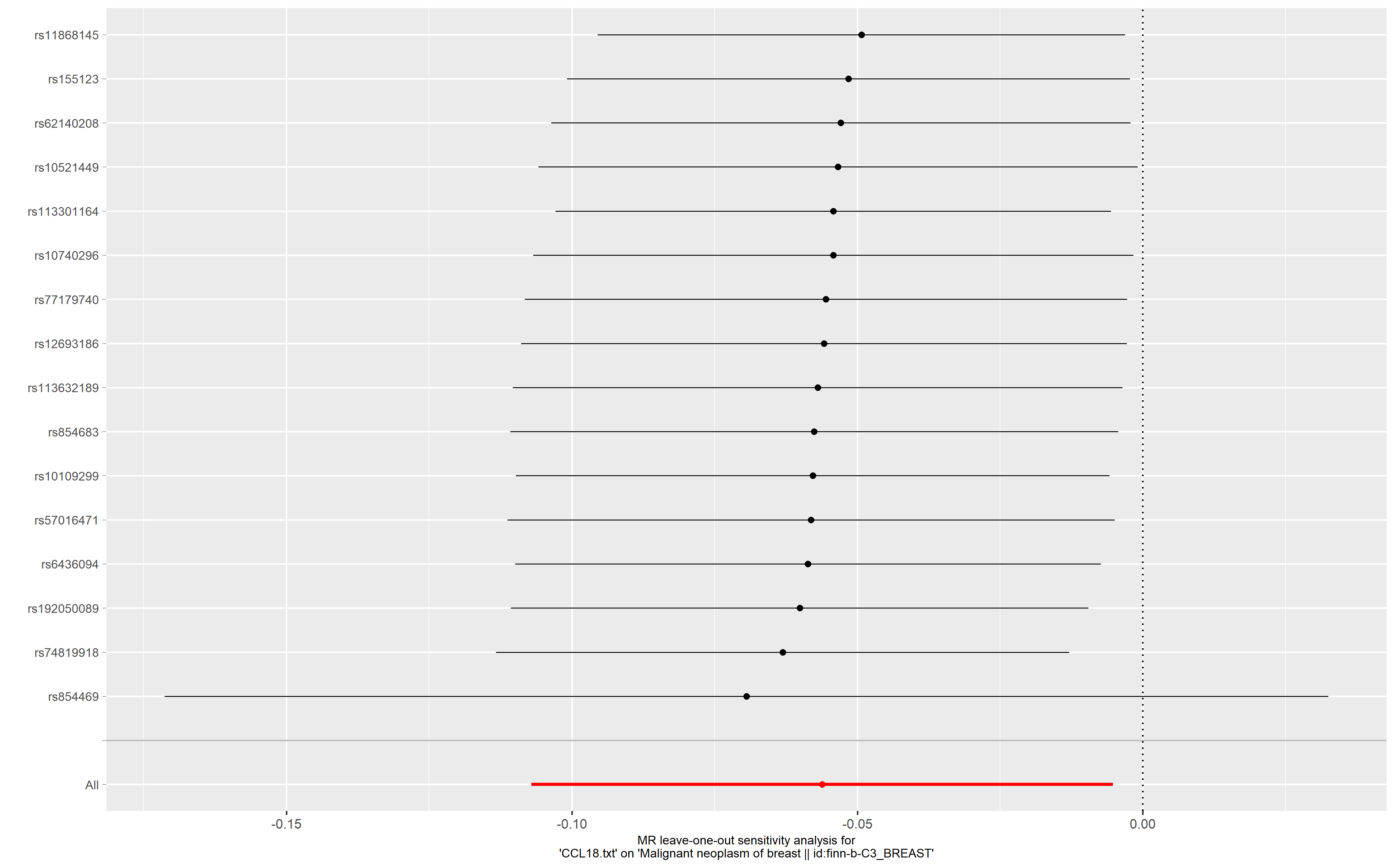


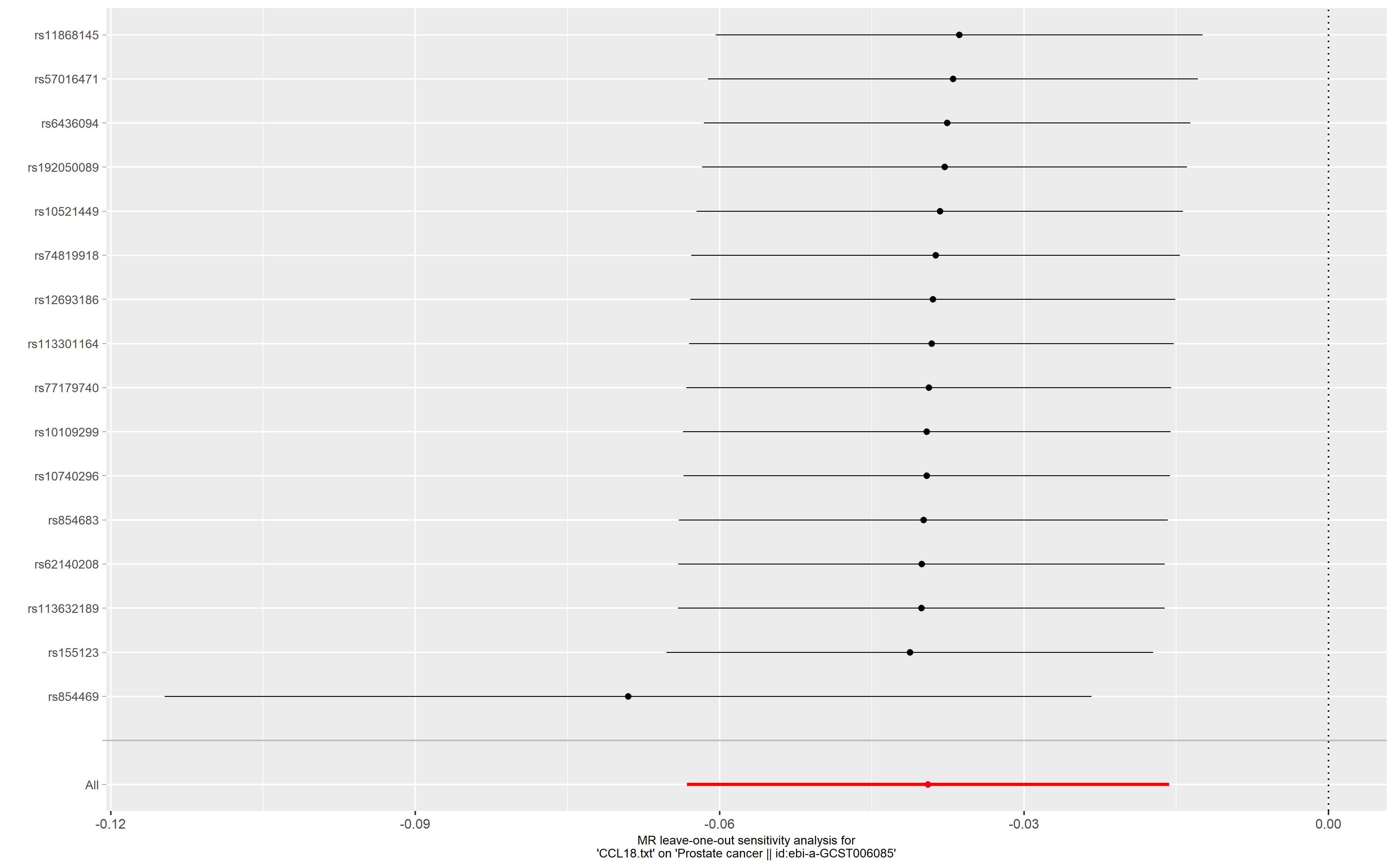


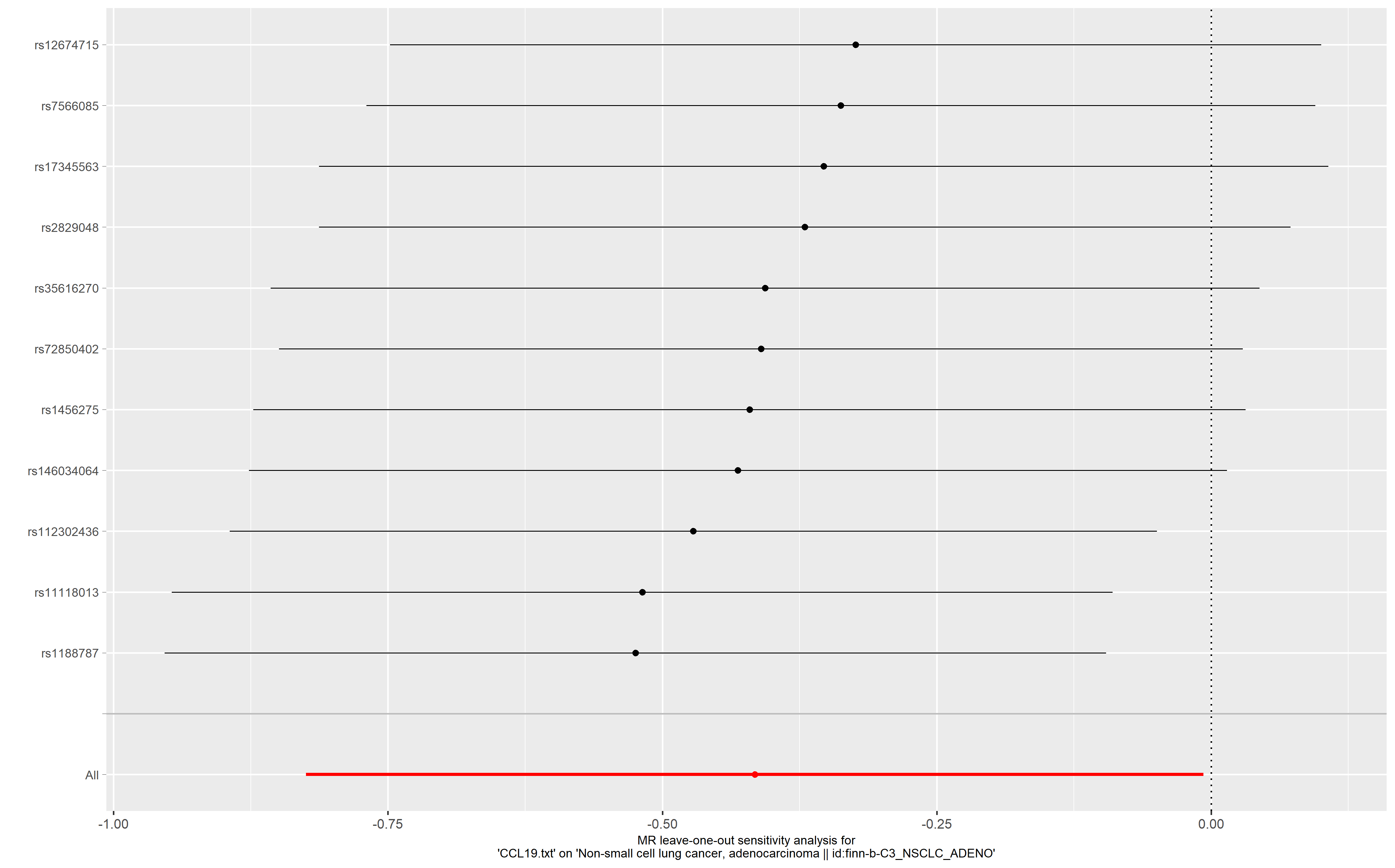


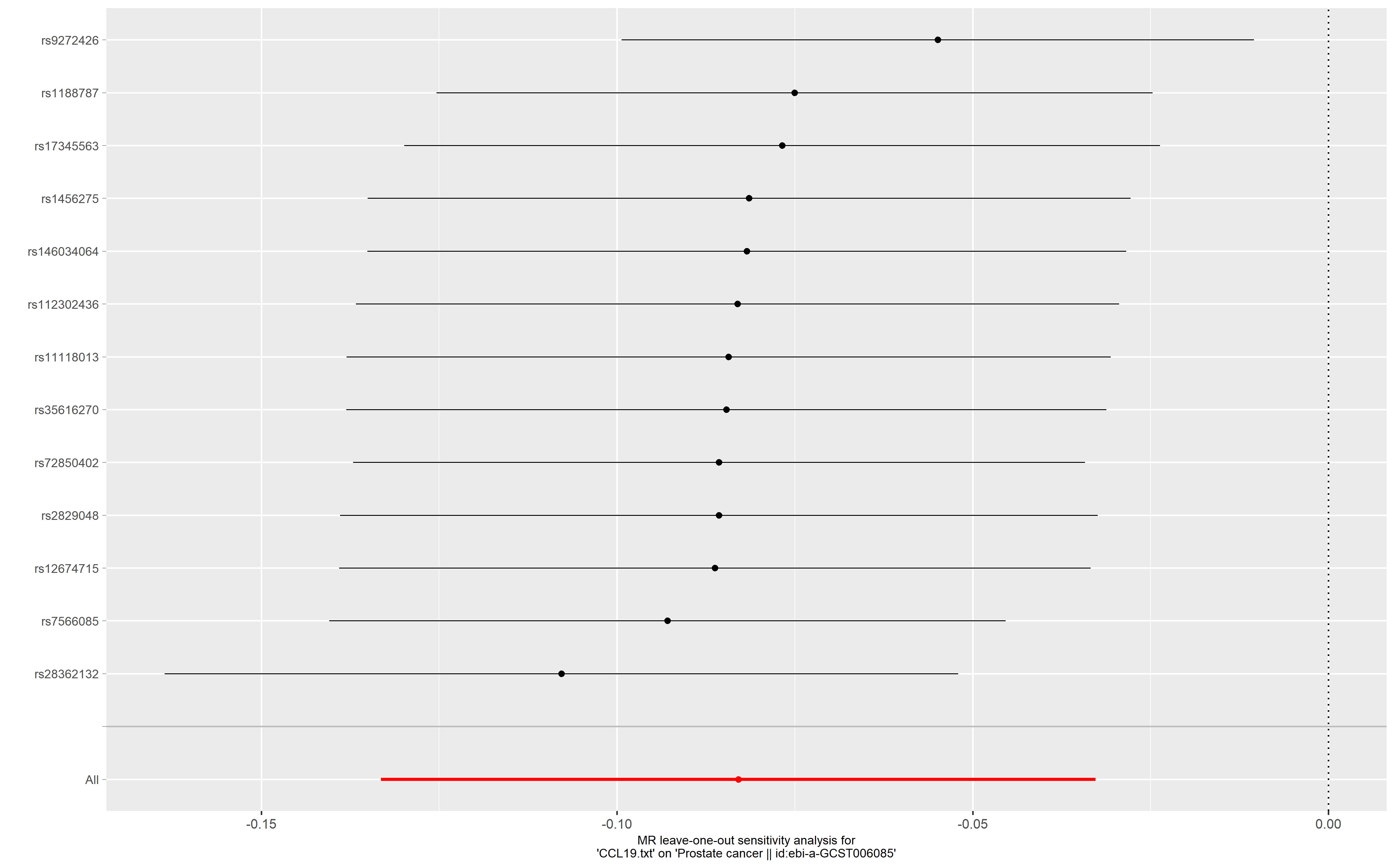


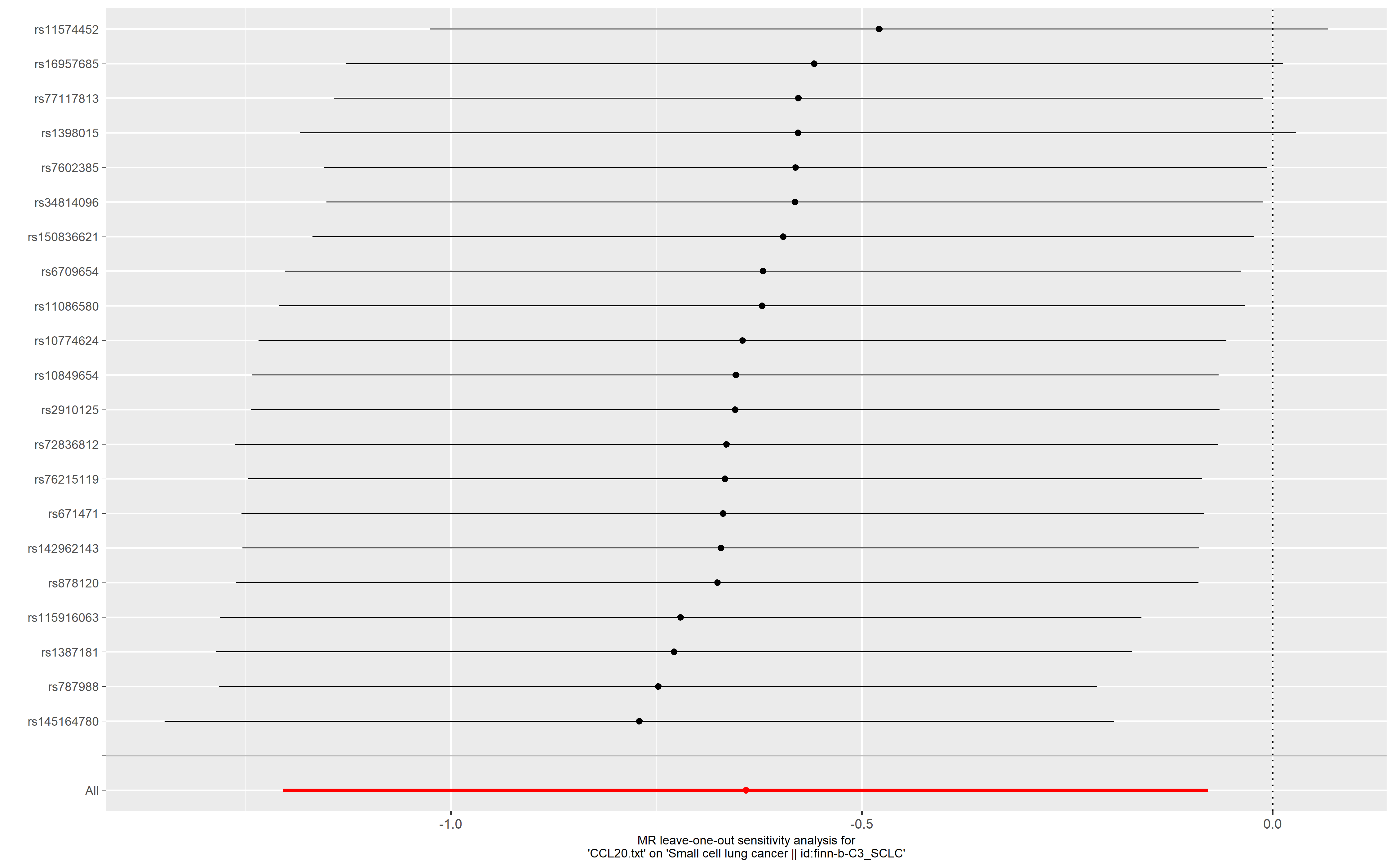


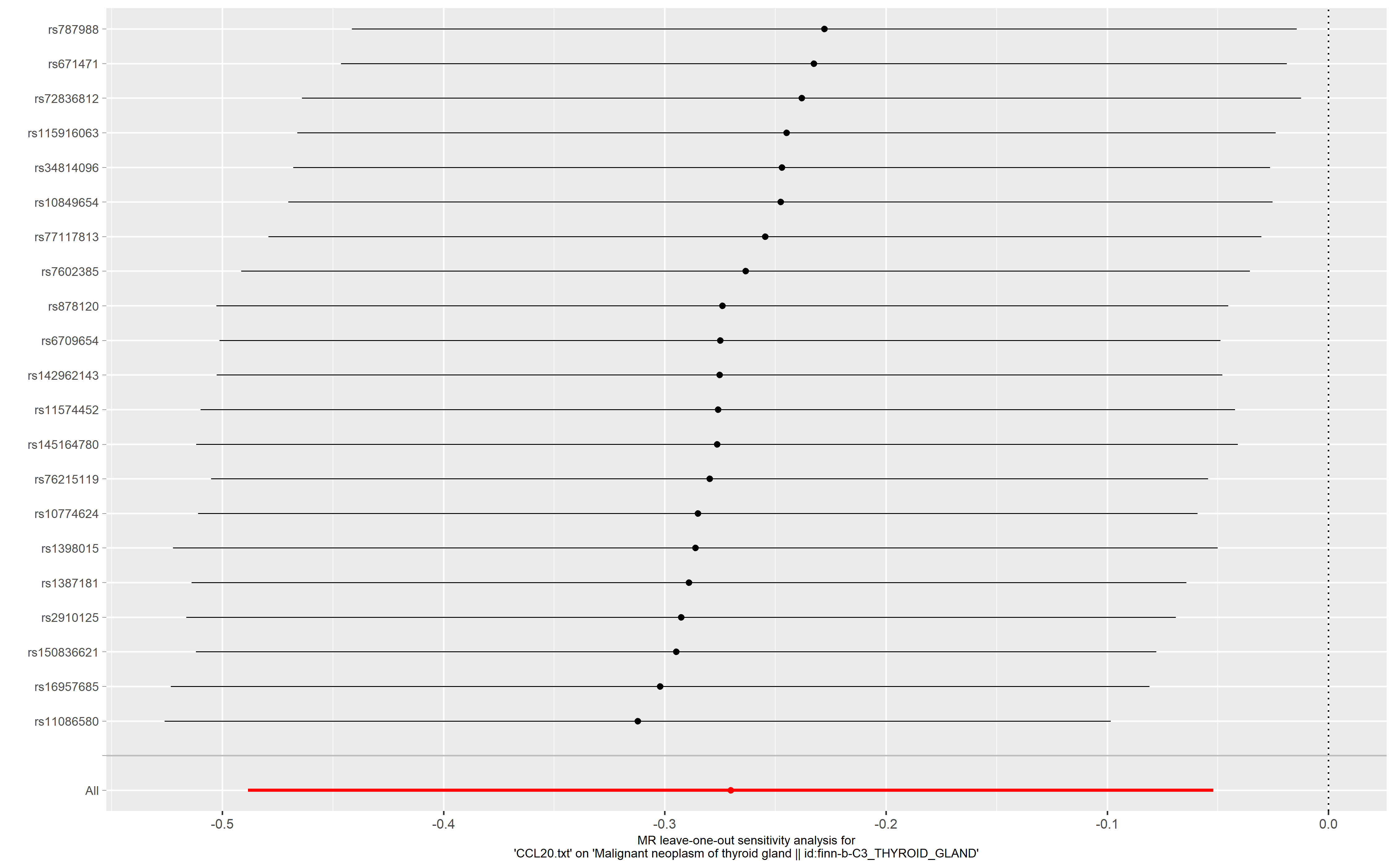


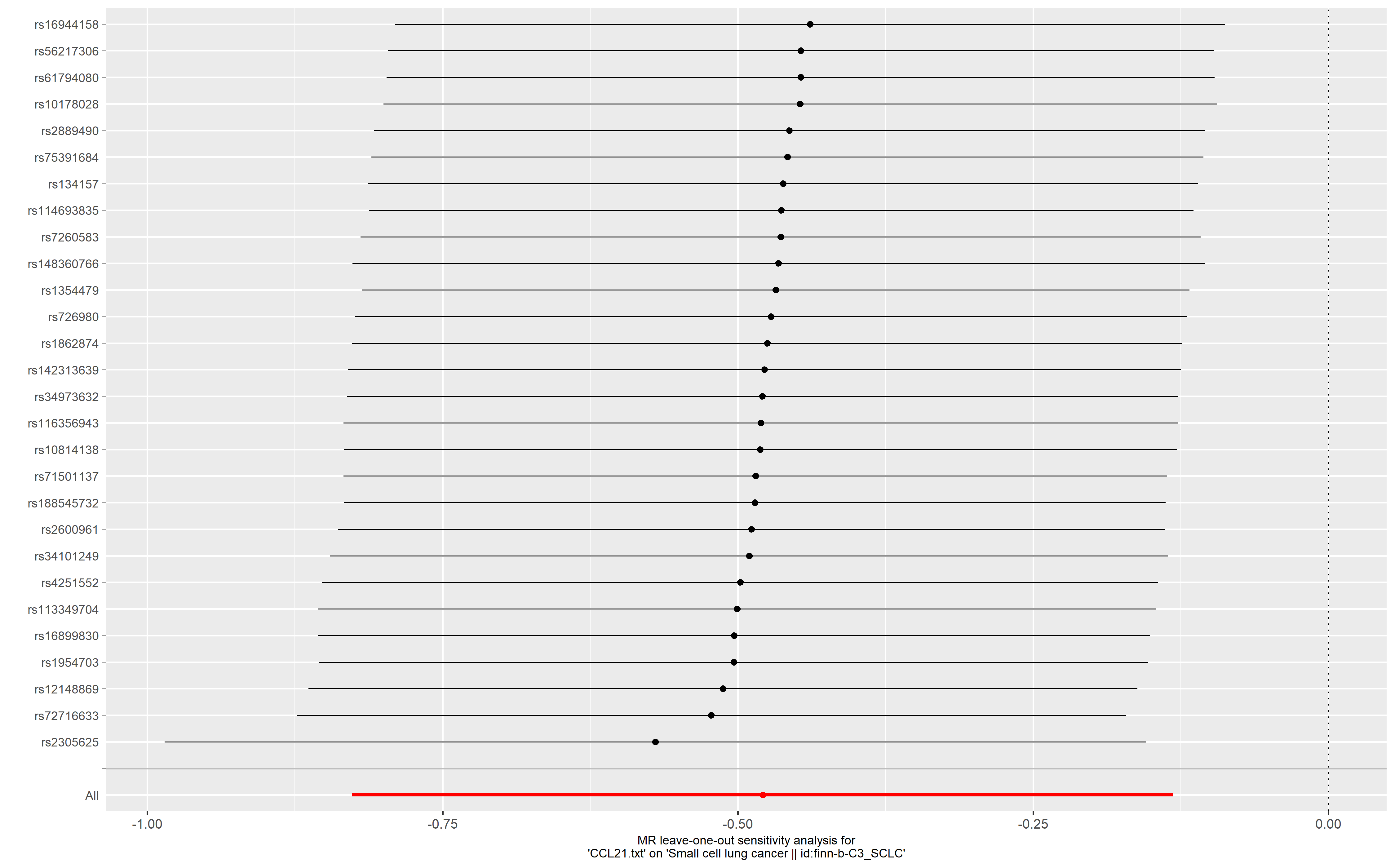


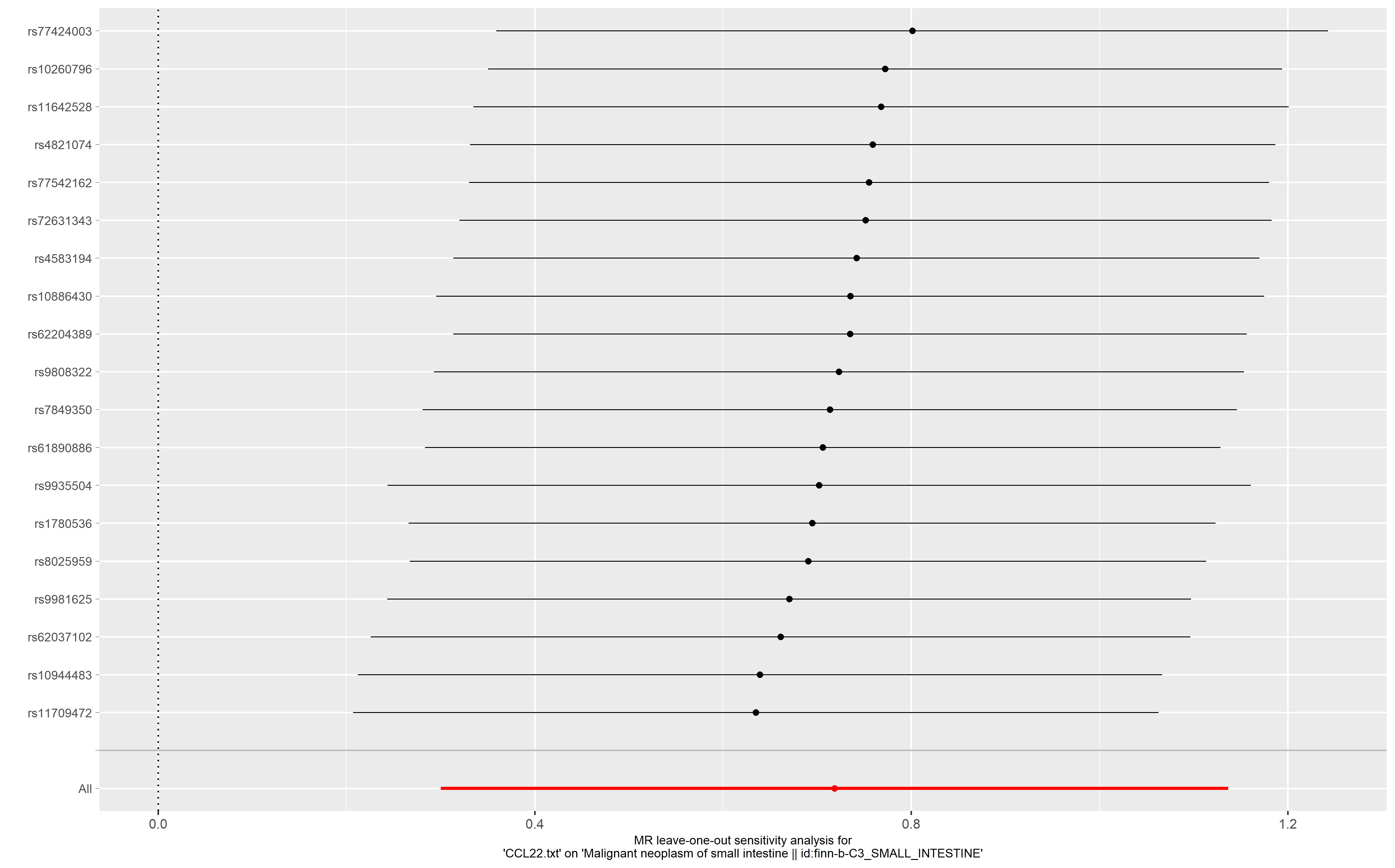


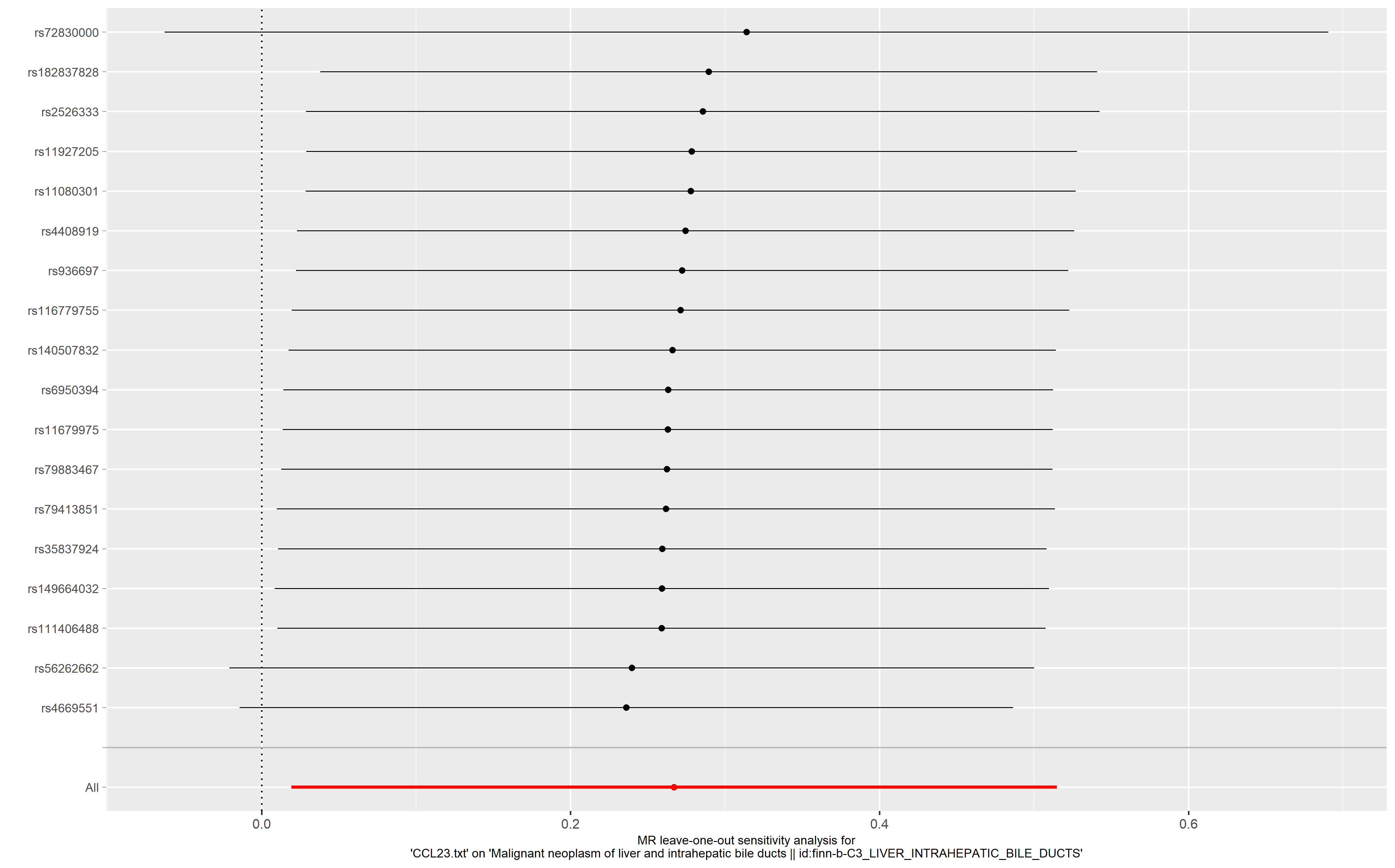


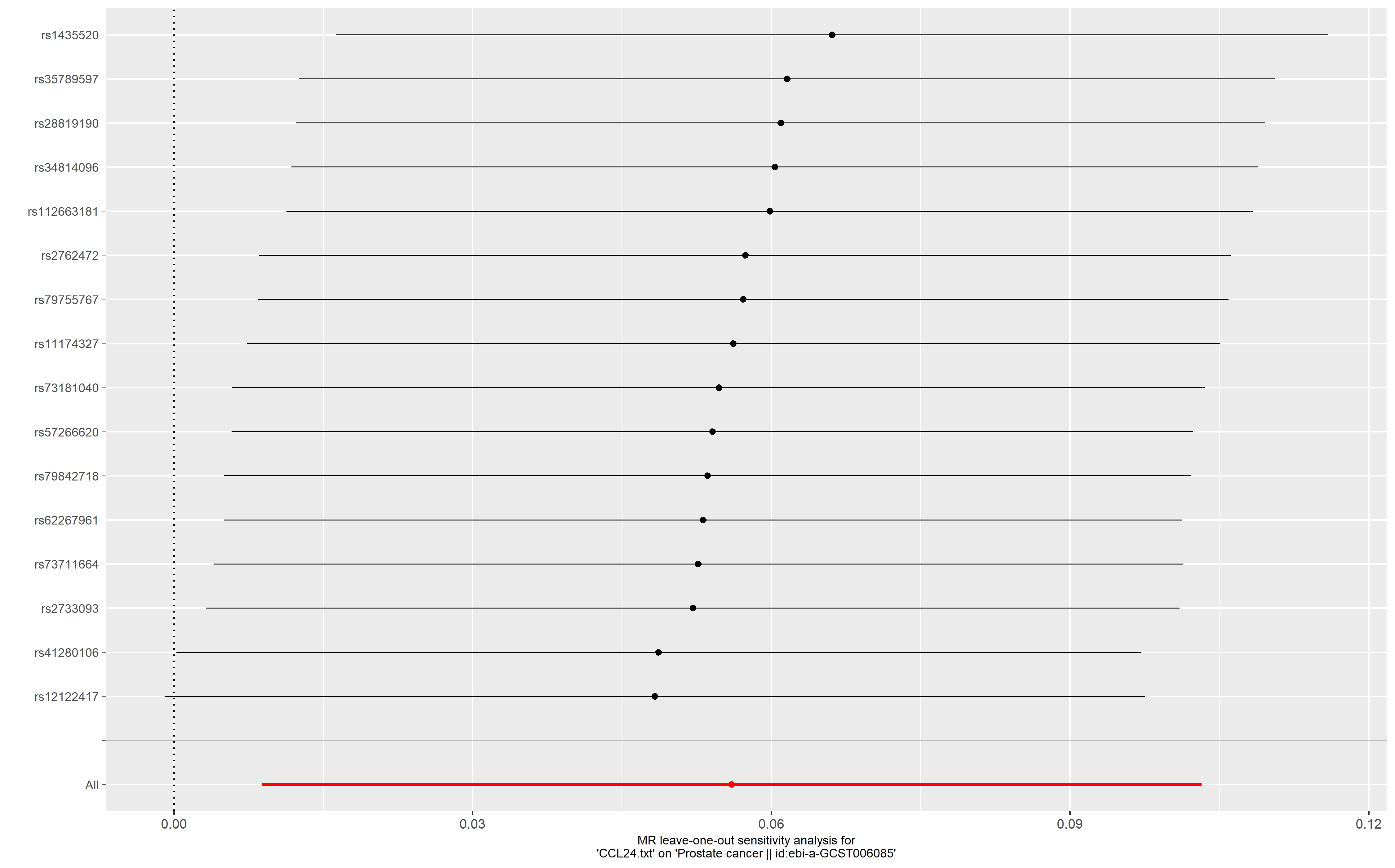


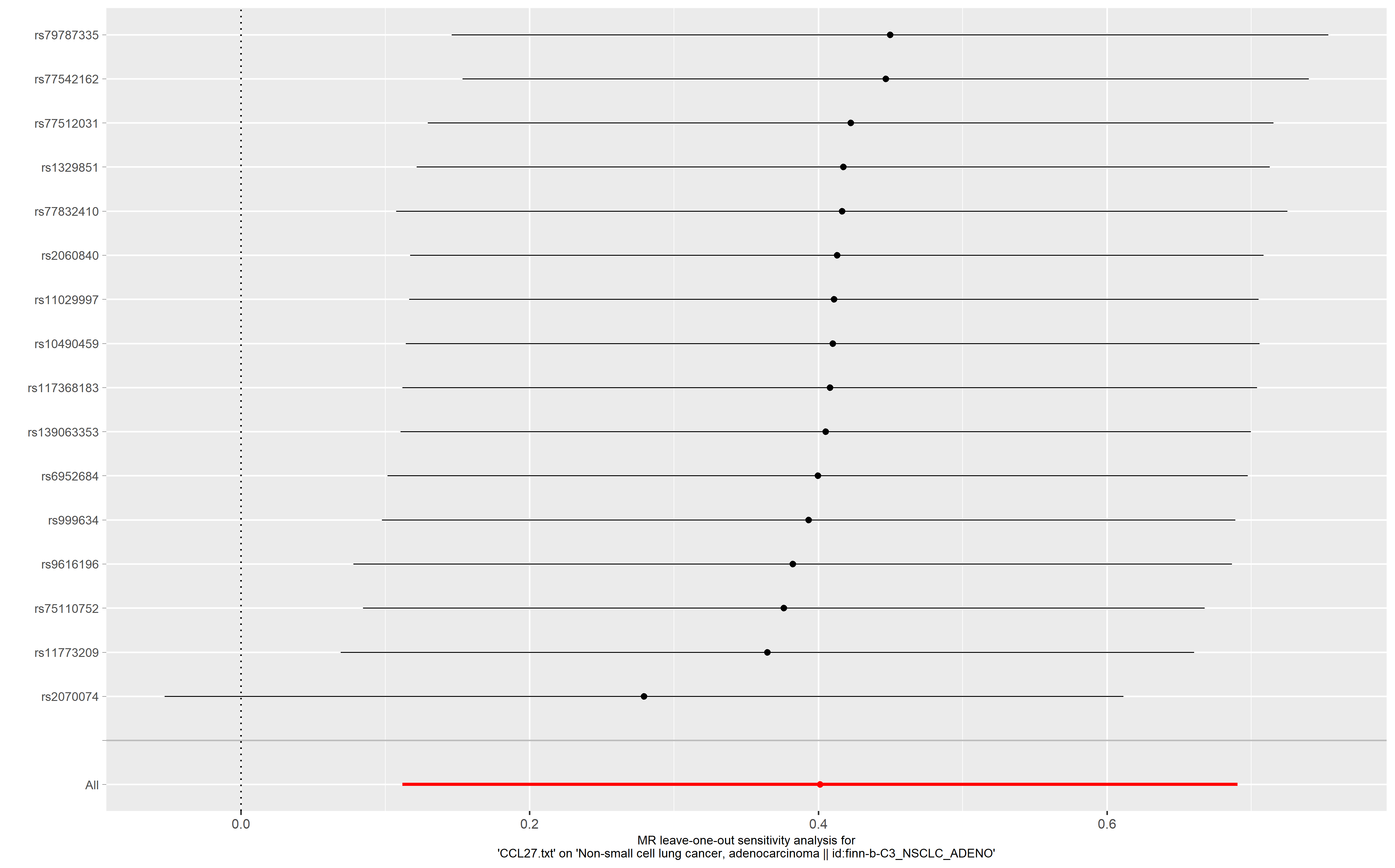


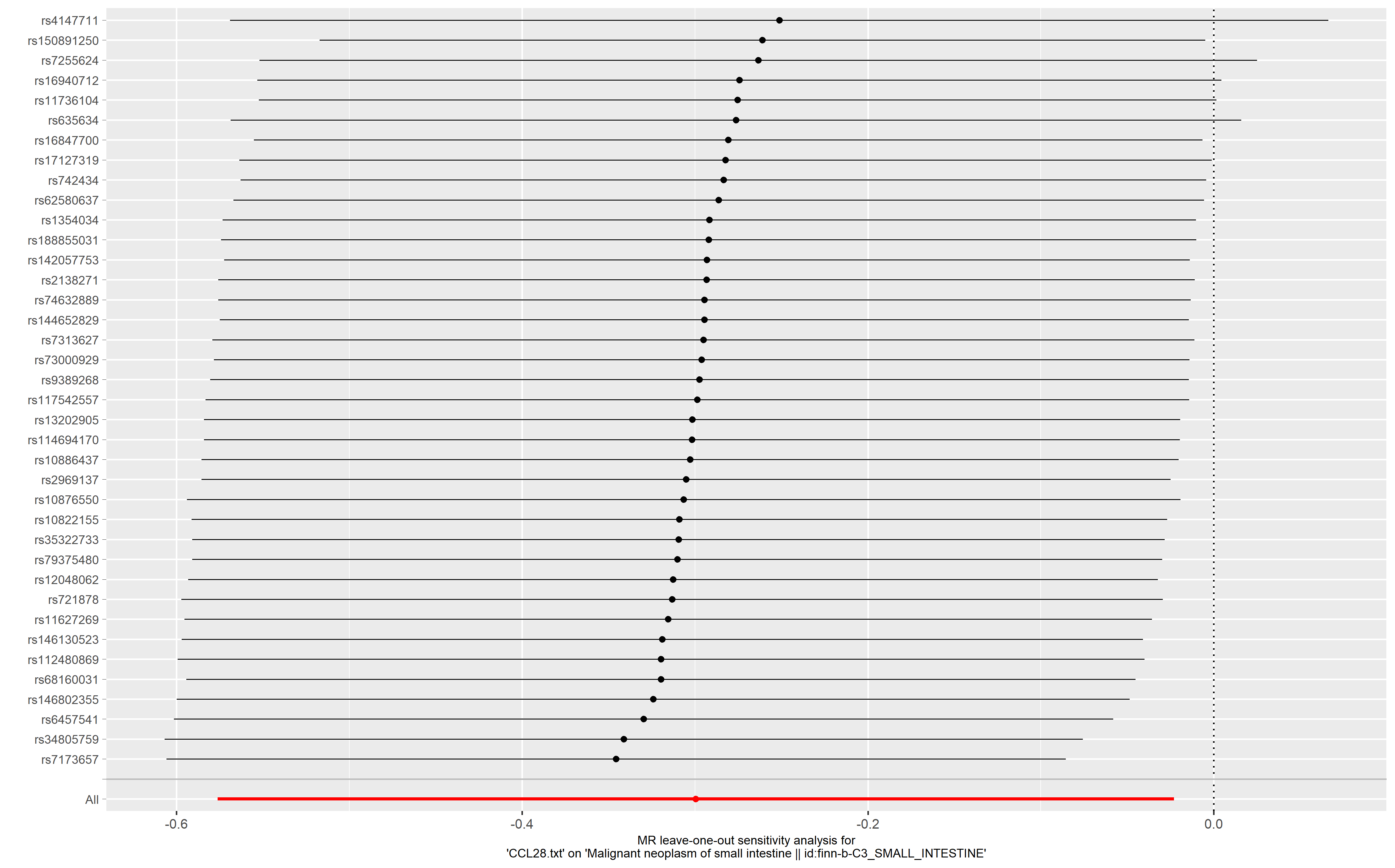


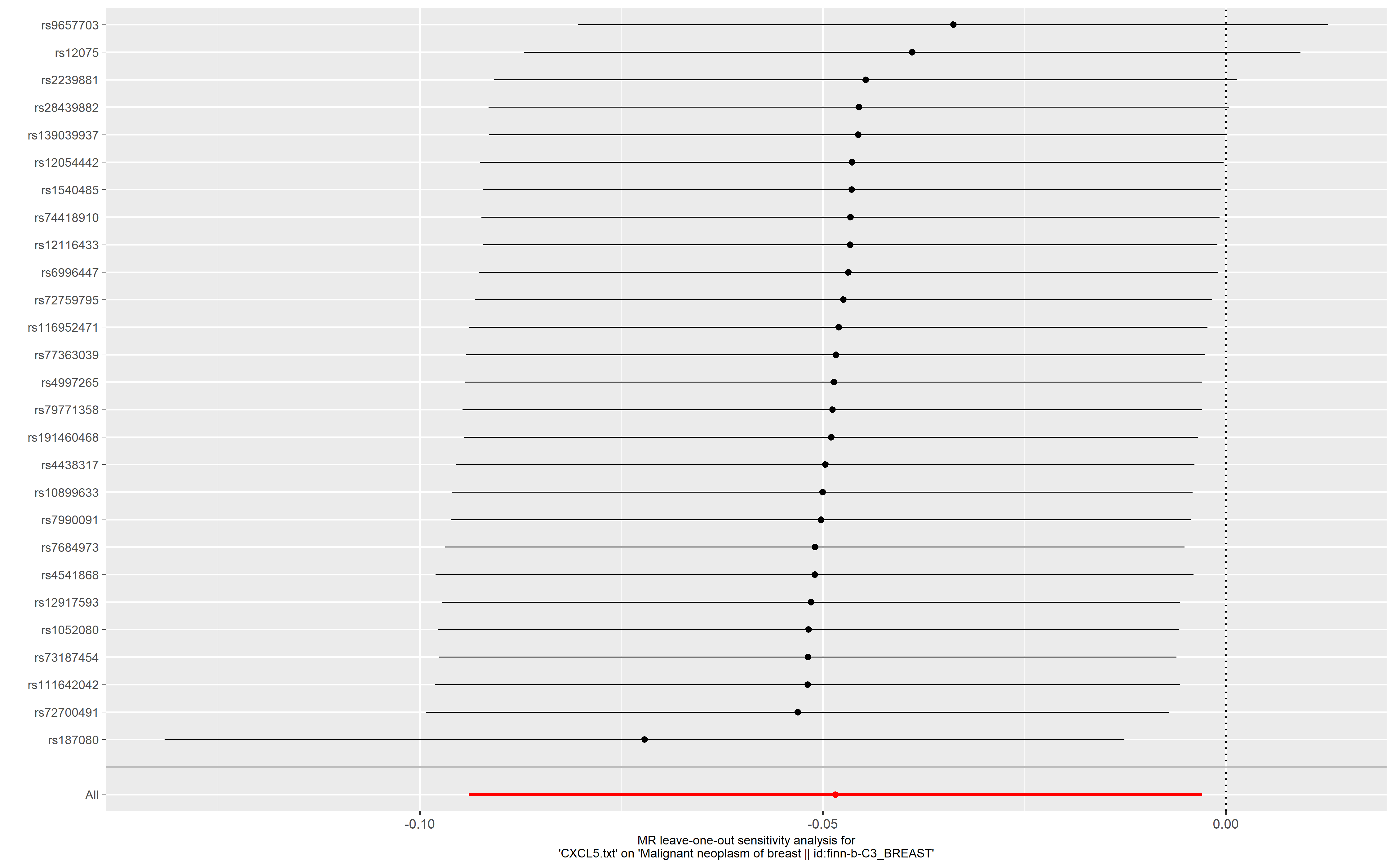


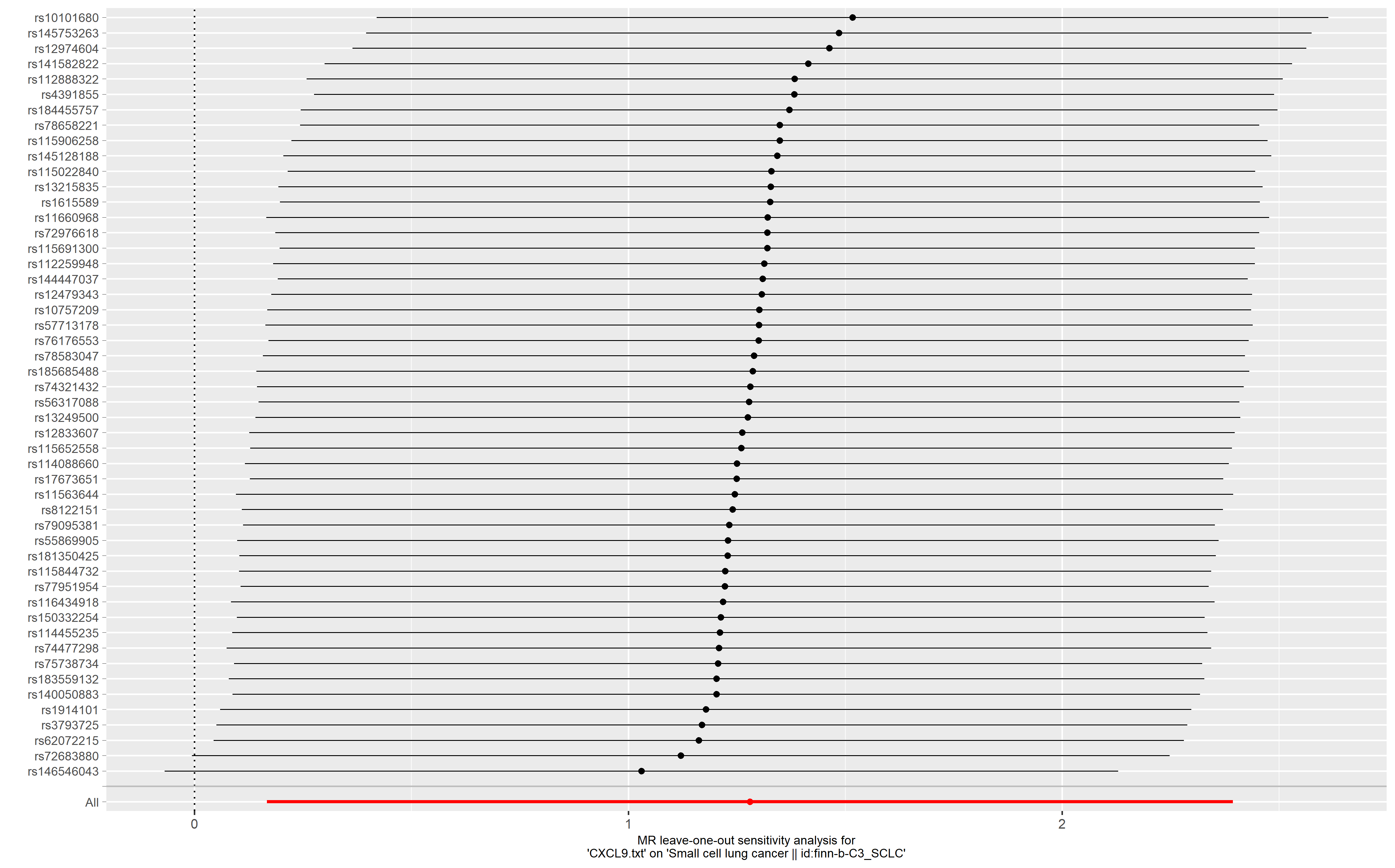


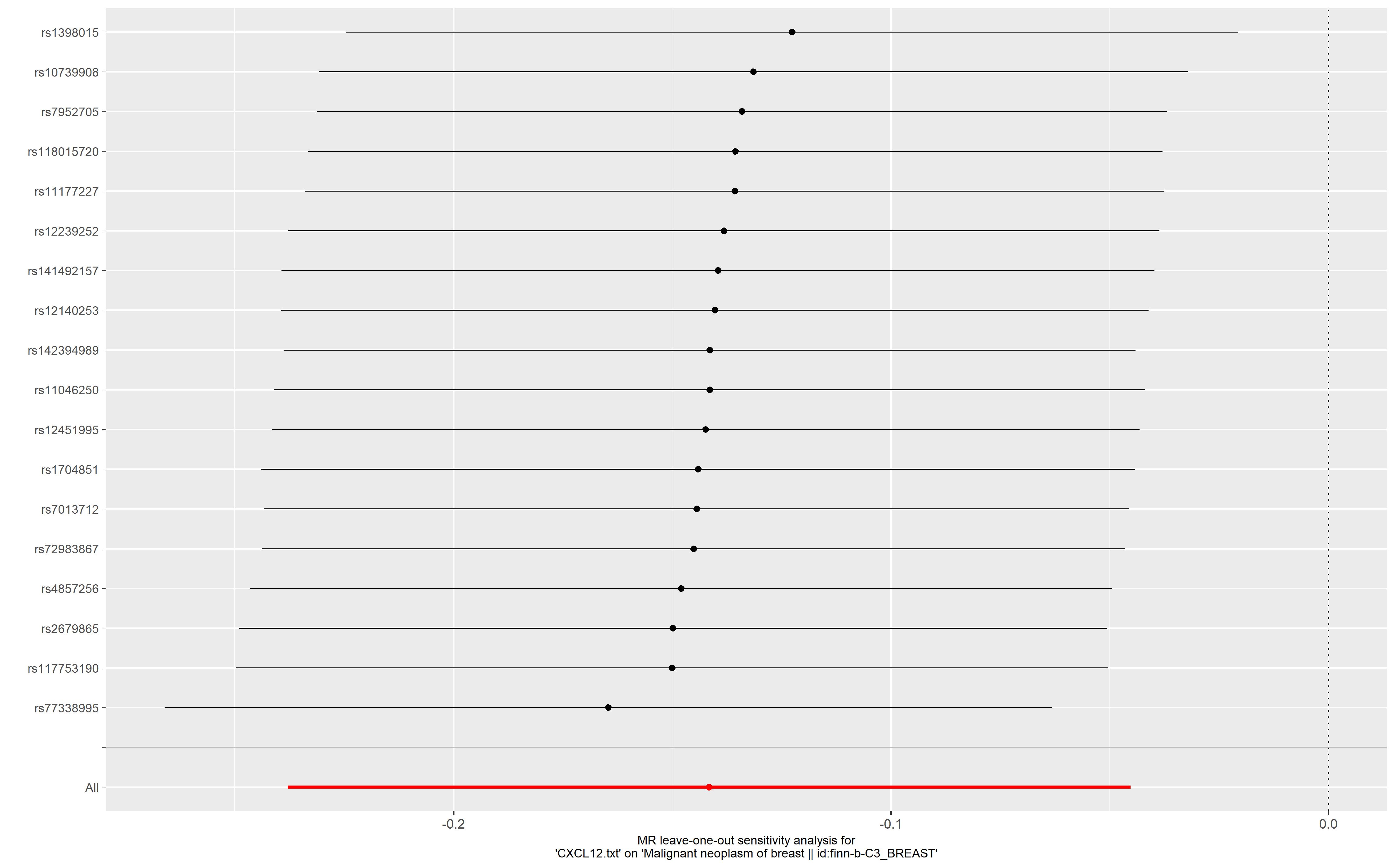


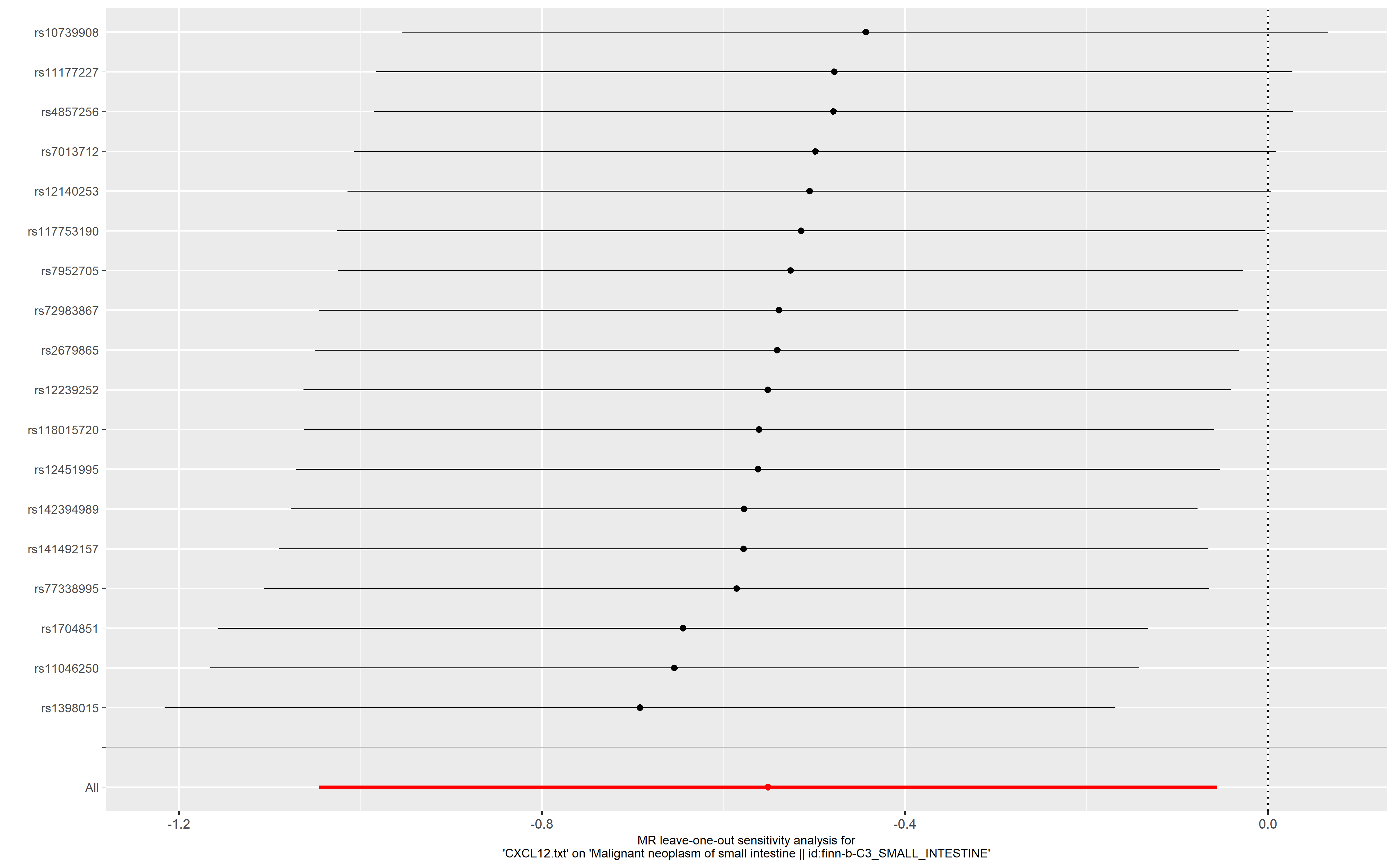


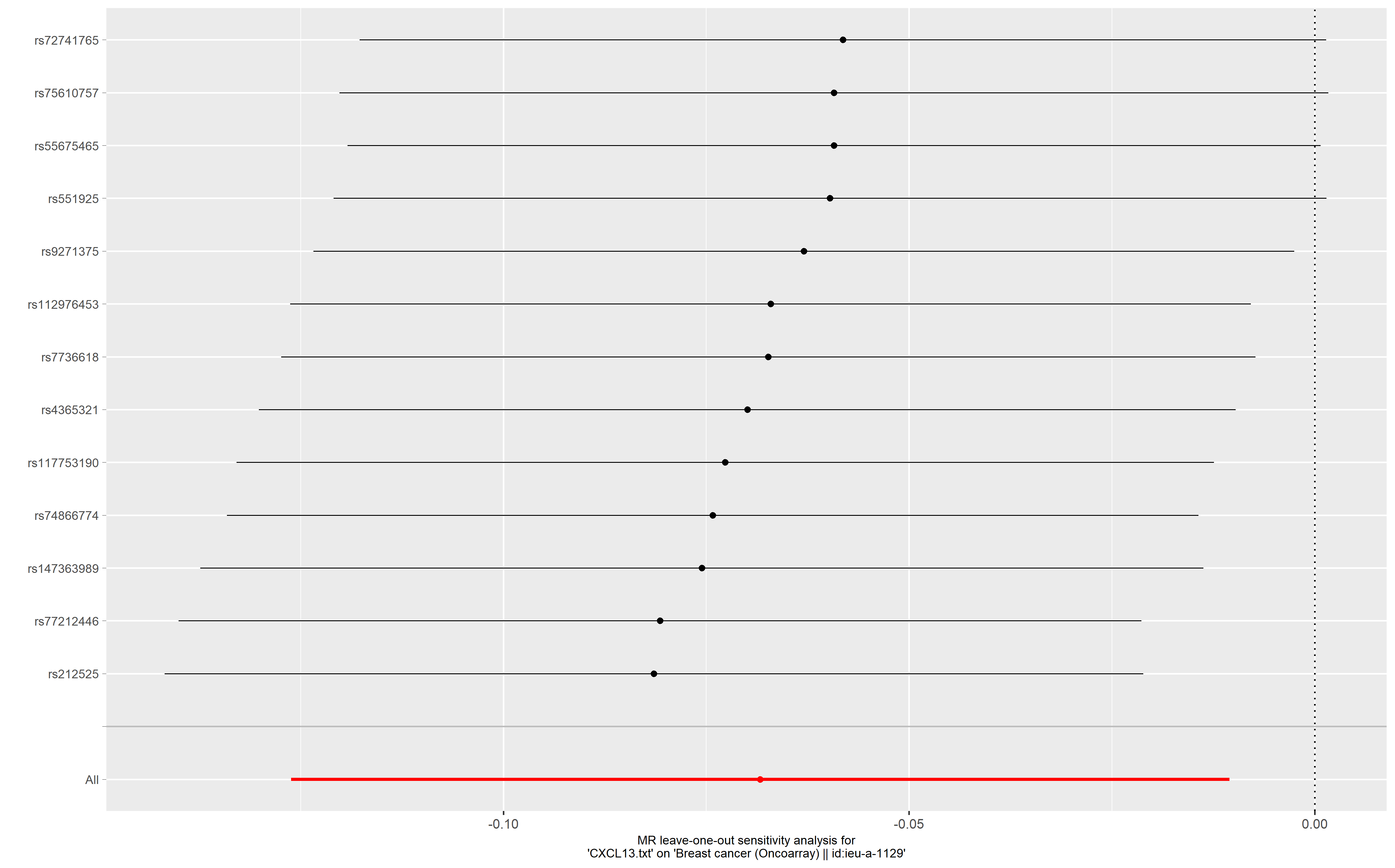


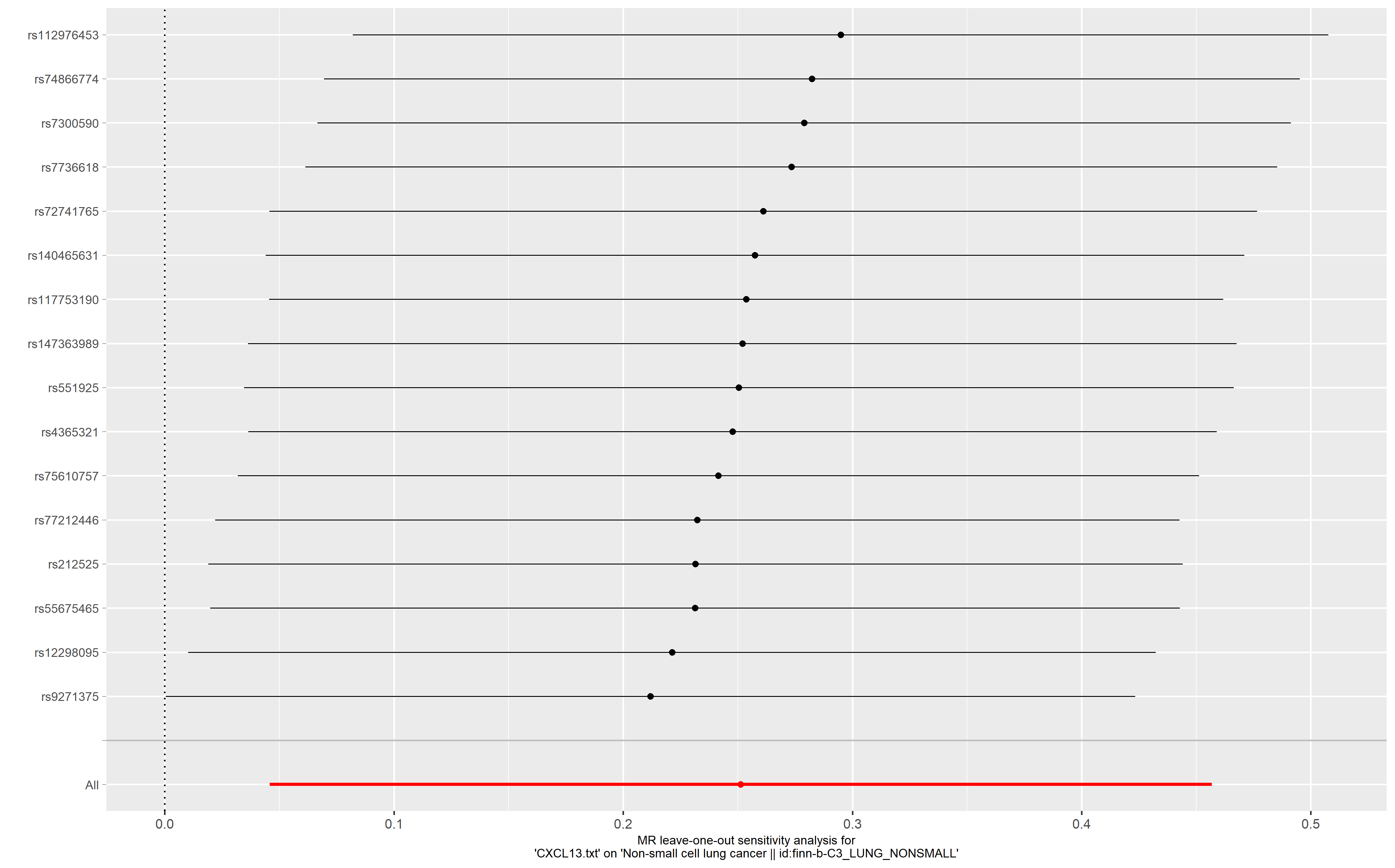


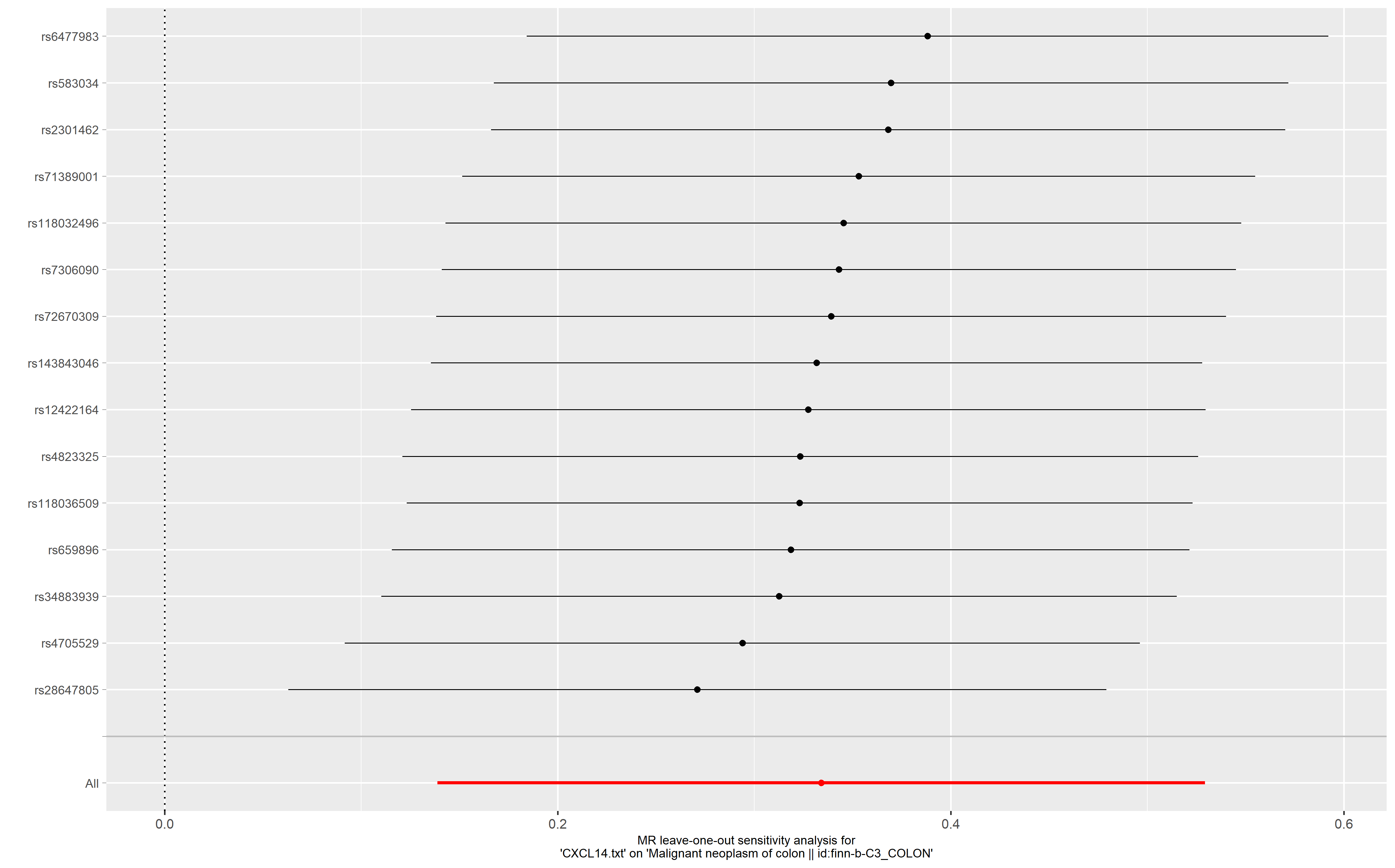


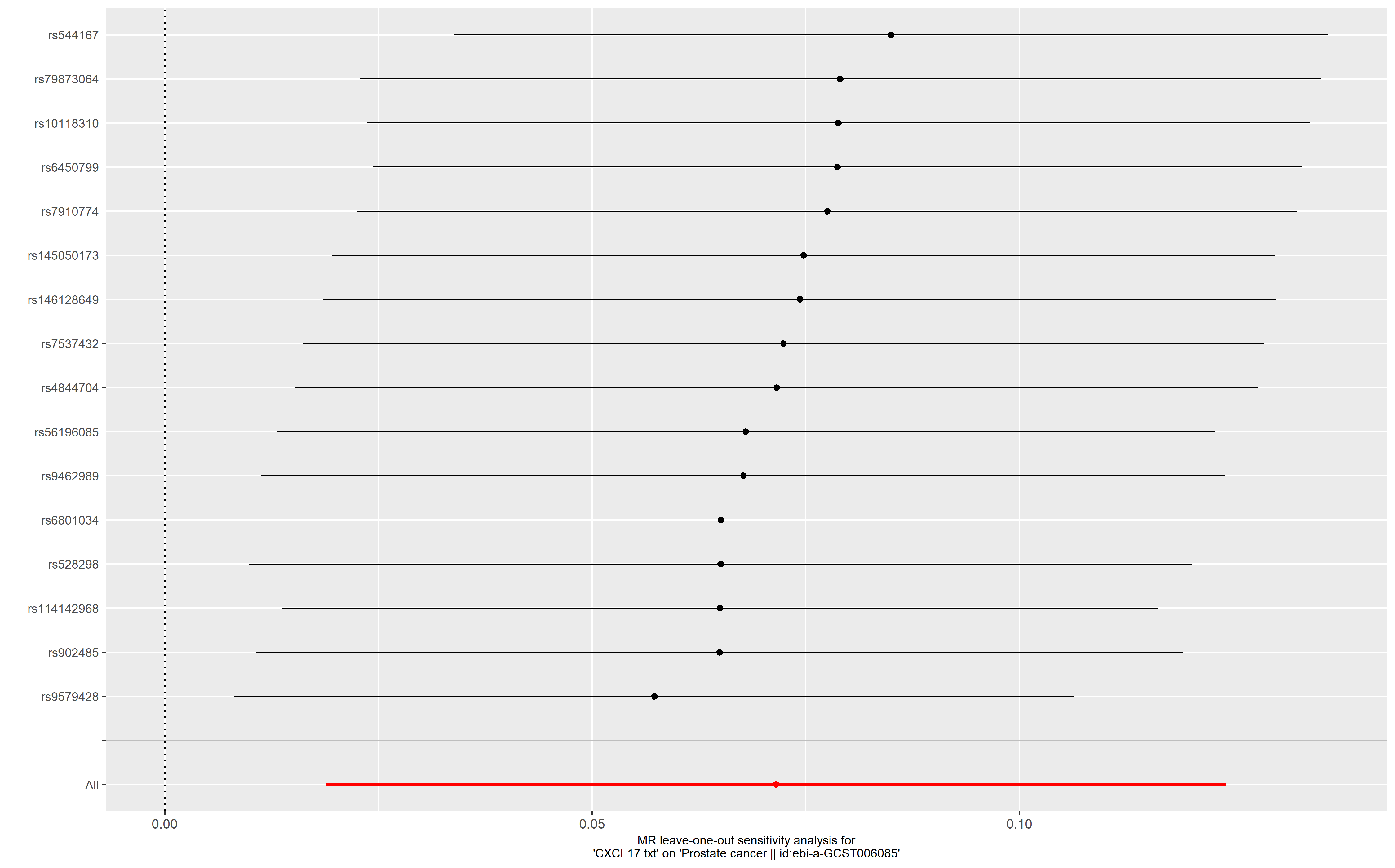

Supplement: Supplementary file 1 [file DataSheet2.zip › Supplementary file/Table 5 Mendelian randomization leave-one-out sensitivity analysis.DOCX]
